# Cryo-EM reveals a nearly complete PCNA loading process and unique features of the human alternative clamp loader CTF18-RFC

**DOI:** 10.1073/pnas.2319727121

**Published:** 2024-04-26

**Authors:** Qing He, Feng Wang, Michael E. O’Donnell, Huilin Li

**Affiliations:** ^a^Department of Structural Biology, Van Andel Institute, Grand Rapids, MI 49503; ^b^DNA Replication Laboratory, The Rockefeller University, New York, NY 10065; ^c^HHMI, The Rockefeller University, New York, NY 10065

**Keywords:** clamp loader, CTF18-RFC, PCNA, DNA replication, sliding clamp

## Abstract

The DNA sliding clamp PCNA is a multipurpose platform for DNA polymerases and many other proteins involved in DNA metabolism. The topologically closed PCNA ring needs to be cracked open and loaded onto DNA by a clamp loader, e.g., the well-studied pentameric ATPase complex RFC (RFC1-5). The CTF18-RFC complex is an alternative clamp loader found recently to bind the leading strand DNA polymerase ε and load PCNA onto leading strand DNA, but its structure and the loading mechanism have been unknown. By cryo-EM analysis of in vitro assembled human CTF18-RFC–DNA–PCNA complex, we have captured seven loading intermediates, revealing a detailed PCNA loading mechanism onto a 3′-ss/dsDNA junction by CTF18-RFC. Interestingly, the alternative loader has evolved a highly mobile CTF18 AAA+ module likely to lower the loading activity, perhaps to avoid competition with the RFC and to limit its role to leading strand clamp loading. To compensate for the lost stability due to the mobile AAA+ module, CTF18 has evolved a unique β-hairpin motif that reaches across RFC2 to interact with RFC5, thereby stabilizing the pentameric complex. Further, we found that CTF18 also contains a separation pin to locally melt DNA from the 3′-end of the primer; this ensures its ability to load PCNA to any 3′-ss/dsDNA junction, facilitated by the binding energy of the E-plug to the major groove. Our study reveals unique structural features of the human CTF18-RFC and contributes to a broader understanding of PCNA loading by the alternative clamp loaders.

## Significance

CTF18-RFC, an alternative clamp loader, localizes to replication forks during the S phase and forms a complex with DNA polymerase ε (Polε). CTF18-RFC has recently emerged as a specific clamp loader for leading strand DNA synthesis. Beyond a role in leading strand synthesis, CTF18-RFC contributes to establishing cohesion between sister chromatids, the DNA replication cell cycle checkpoint pathway, and in genomic stability, especially at trinucleotide repeats. Using cryogenic electron microscopy, this report determines seven structures of the human CTF18-RFC–PCNA complex with or without DNA, providing a molecular picture of a nearly complete PCNA loading process by the CTF18-RFC loader.

Sliding clamps encircle and slide along duplex DNA, acting as a mobile platform to localize DNA polymerase or other binding partners, to promote processive DNA synthesis and regulate the activities of many proteins involved in DNA repair (1–3). Proliferating cell nuclear antigen (PCNA) is the eukaryotic sliding clamp that functions as a hub protein and plays critical roles in many essential processes, including DNA replication, cell cycle control, nucleotide excision repair, break-induced replication, mismatch repair, and chromatin assembly (4–8).

Loading of a sliding clamp onto DNA is accomplished by clamp loaders (9, 10), which belong to the AAA+ family (ATPases associated with various cellular activities) (11, 12). While some AAA+ family members assemble into a hexameric structure to translocate on a nucleic acid or a peptide substrate in an ATP-dependent manner (13–19), clamp loaders adopt a distinct pentameric structure (2). The most studied clamp loader is the RFC complex which is composed of the largest subunit RFC1 (Rfc1 in yeast) and four smaller subunits RFC2-5 (yeast Rfc2-5) (20–22). The arrangement of the five subunits (A-B-C-D-E) going counterclockwise when viewed down the C-terminal face is structurally the same in yeast and human RFC, Rfc1/A-Rfc4/B-Rfc3/C-Rfc2/D-Rfc5/E in yeast and RFC1/A-RFC2/B-RFC5/C-RFC4/D-RFC3/E in human, respectively (23). The subunit numbers in the pentamer are a bit different due to their migration in SDS gels. For clarity, we also refer here to clamp loader subunits in the A, B, C, D, and E positions. RFC1 (A subunit) can be replaced by other homologs, like CTF18, ATAD5 (Elg1 in yeast), and RAD17 (Rad24 in yeast) to form alternative RFC-like clamp loaders or unloaders (20, 21). Interestingly, the RFC and CTF18-RFC complexes primarily load the PCNA clamp onto DNA, although they also have a low PCNA unloading activity (24–26). In contrast, the ATAD5-RFC (Elg1-RFC in yeast) complex only unloads PCNA from DNA. Unlike all the PCNA loaders/unloaders, RAD17-RFC (yeast Rad24-RFC) loads the 9-1-1 DNA damage checkpoint clamp (human RAD9-HUS1-RAD1; yeast Ddc1-Mec3-Rad17) onto DNA (27–29). Each of the clamp loaders contains an A-gate between the first subunit (A) and the last subunit (E) for specific recognition and clamp loading at a 3′-end or a 5′-end single-stranded/double-stranded (ss/ds) DNA junction (22).

**Fig. 1.**
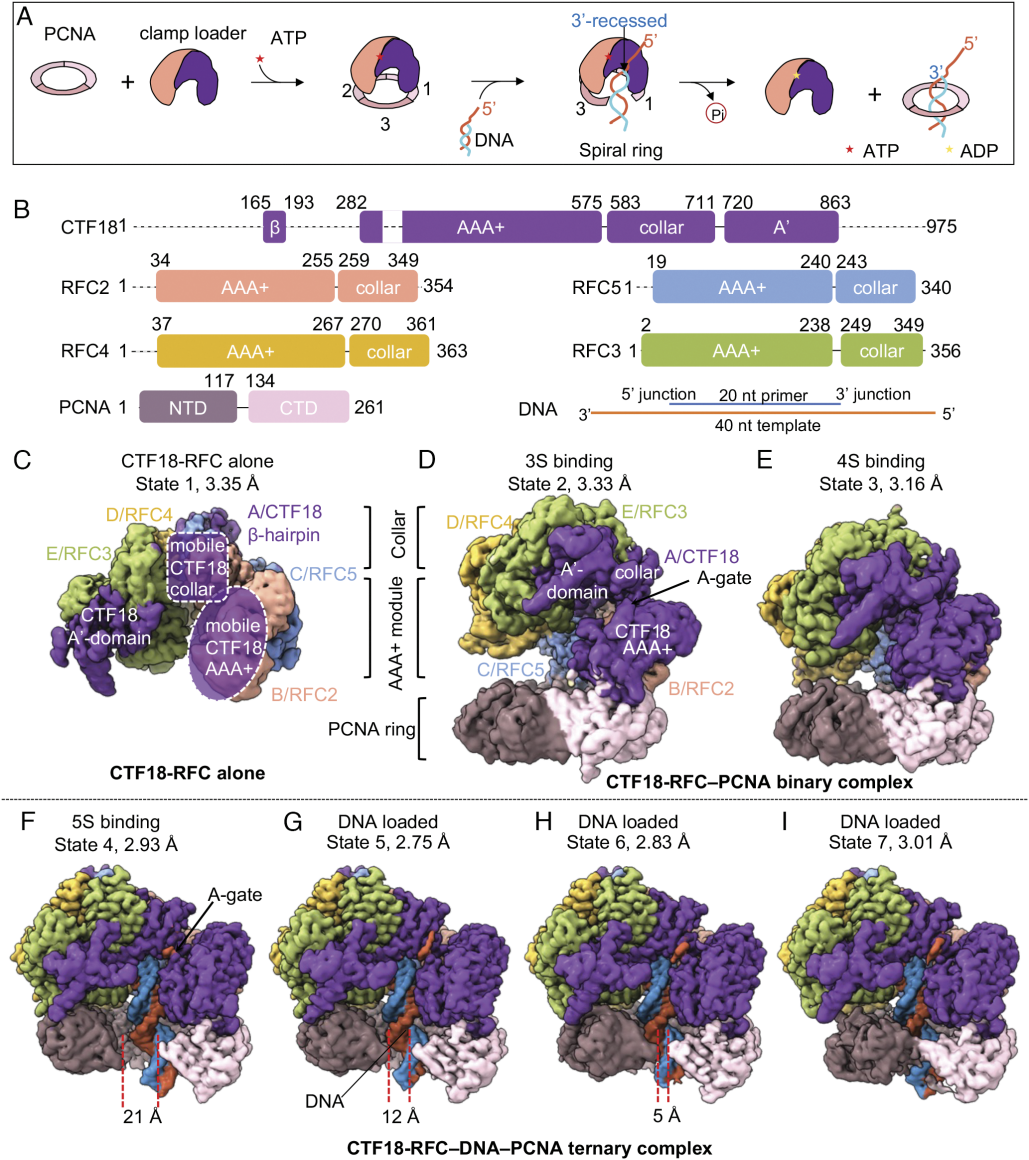
Cryo-EM maps of the human CTF18-RFC alone and complexed with PCNA and DNA. (*A*) Schematic view of the human CTF18-RFC-dependent loading of PCNA onto a 3′-ss/ds DNA junction. (*B*) Domain architectures of CTF18, RFC2-5, PCNA, and DNA substrate. Dashed lines indicate disordered regions. DCC1 and CTF8 are invisible in all maps. (*C*–*I*) EM maps of the CTF18-RFC alone and with PCNA ± DNA. (*C*) In apo, referred to as state 1, (*D*) the binary CTF18-RFC–PCNA map in which 3 clamp loading subunits bind PCNA (3S-binding mode), also referred to as state 2, (*E*) the binary CTF18-RFC–PCNA map in which 4 clamp loading subunits bind PCNA (4S-binding mode) referred to as state 3, (*F*–*I*) the ternary CTF18-RFC–DNA–PCNA complexes (5S-binding mode) (*F*) state 4: (*G*) state 5, (*H*) state 6 and, (*I*) and the closed-ring state 7. CTF18-RFC contains an upper collar tier and a lower AAA+ module tier conserved among the clamp loaders. Individual CTF18 domains are labeled in *C*. The maps are colored by individual subunits as in *B*. DNA strands are individually colored by orange (template) and blue (primer). The PCNA gap is indicated by two dashed red lines.

Pioneering studies on crystal structures of clamp loaders of eukaryotes, archaea, bacteria, and the T4 bacteriophage, revealed key insights into the clamp loading mechanism of clamp loaders (30–34). The clamp loading mechanism contains three basic steps (Fig. 1*A*): 1) an ATP-bound clamp loader engages a clamp; 2) the ATP binding energy drives clamp ring opening and converts the clamp to a right-handed spiral by templating on the spiral AAA+ tier of the loader, leading to DNA entry into the inside of both the open clamp and the central chamber of the clamp loader pentamer, 3) the clamp recloses to topologically encircle the DNA and separates from the clamp loader (21, 22).

The five subunits (Rfc1-5) of yeast RFC assemble head-to-tail into a two-tiered structure, with the bottom tier containing five AAA+ ATPase modules plus the C-terminal A’ domain of Rfc1, and the top tier containing five α-helical collar domains arranged in a circle and hold the pentameric structure together (2, 9). RFC-ATP binds PCNA to crack open the PCNA ring, allowing DNA to enter PCNA and the inner chamber of RFC, and then DNA binding induces ATP hydrolysis that separates RFC from PCNA encircling dsDNA (33, 35, 36). However, the loading process is likely highly dynamic, and EM analysis may find more intermediates. Recently, cryo-EM analyses have revealed that yeast Rad24-RFC and human RAD17-RFC contain an external “shoulder” site that binds 5′-recessed DNA (5′ ss/ds DNA) to facilitate clamp loading (27–29). An analogous 5′ ss/ds site was then found to exist in RFC as well (35–39).

Uniquely, CTF18-RFC is a heptamer with two additional non-ATPase subunits DCC1 and CTF8 (Fig. 1*B*) (24–26). In vitro assays have shown that Ctf18-RFC only loads PCNA onto a 3′-recessed DNA, like RFC, but the loading activity is lower than that of RFC (25, 26). Ctf18-RFC was also reported to possess a potent PCNA unloading activity in vitro, although such activity has yet to be determined in vivo (25). A reconstituted functional human replisome assay showed that CTF18-RFC accelerates leading strand DNA synthesis in the presence of PCNA (40). Beyond clamp loading, yeast Ctf18-RFC was shown to contribute to the DNA replication checkpoint by activating the Rad53 checkpoint kinase when a replication fork stalls (41). Ctf18-RFC both enhances and maintains the balance of PCNA levels at the replication fork, which affect the recruitment of Eco1 cohesion acetyltransferase to establish sister chromatid cohesion (42, 43). Ctf18-RFC also functions with Scc2 to load cohesin de novo (44). Furthermore, the yeast Ctf18-RFC has also recently been shown to promote genomic stability, particularly in the fragile CAG/CTG trinucleotide repeat regions (45).

Recent structural work has shown that DCC1, CTF8, and the C-terminal tail of CTF18 form a “hook module” that is flexibly linked to the ATPase pentamer composed of CTF18-RFC2-5 (46–48). This hook module interacts with the catalytic domain of the leading strand DNA polymerase ε (Polε), thereby targeting CTF18-RFC to the leading strand (26, 46–49). Because the Ctf18-RFC structure was unknown, it was not understood whether the loading mechanism of RFC generalizes to CTF18-RFC and the structural basis for their functional differences. In this cryo-EM study, we reconstituted the human CTF18-RFC–DNA–PCNA complex in vitro and captured seven intermediate states in the PCNA loading cycle by CTF18-RFC, including an apo state of CTF18-RFC before it encounters a clamp, two states of CTF18-RFC bound to PCNA, and four states of CTF18-RFC and PCNA interacting with the 3′-ss/dsDNA junction. These structures reveal detailed interactions between CTF18-RFC, PCNA, and DNA, a series of conformational changes during the dynamic PCNA loading process by CTF18-RFC, and a separation pin in the CTF18 collar domain that unwinds DNA from the 3′-end of the primer strand. This study provides a comprehensive structural and mechanistic understanding of PCNA loading mediated by the human CTF18-RFC complex.

## Results

### Cryo-EM Captures Seven Intermediates of CTF18-RFC Loading PCNA onto DNA.

We recombinantly expressed human CTF18-RFC composed of CTF18, RFC2-5, DCC1, and CTF8 in insect cells (*Method Summary* and *SI Appendix*, Fig. S1*A*). Given that the external shoulder 5′ ss/ds DNA site was found in both RFC and Rad24RFC, we considered that CTF18-RFC may contain an external shoulder DNA site. Thus, we used a double-tailed DNA substrate having both 3′- and 5′-ss/ds DNA junctions (Fig. 1*B* and *SI Appendix*, Fig. S1*B*), which we used previously to find the external 5′ DNA sites in Rad24-RFC and RFC (27, 37). We mixed purified CTF18-RFC, PCNA, and DNA at a molar ratio of 1:3:10 in the presence of 0.5 mM ATPγS, a slowly hydrolyzable ATP analog. Two-dimensional classification of cryo-EM images revealed well-defined particles and the successful assembly of the clamp-clamp loader complexes (*SI Appendix*, Fig. S1 *C* and *D*). Subsequent 3D classification and multiple rounds of refinement resulted in seven 3D EM maps (*SI Appendix*, Figs. S2 and S3). In overview, we observe complexes having either 3, 4, or all 5 clamp loader subunits bound to the PCNA, which we refer to as 3S-, 4S-, and 5S-binding modes. The seven distinctive states involve differences in association with DNA, PCNA, or size of gap in PCNA. These states, summarized in Fig. 1, are CTF18-RFC alone in the apo state (state 1) at an overall resolution of 3.35 Å (Fig. 1*C*), a CTF18-RFC–PCNA binary complex having three clamp loader subunits bound to a closed planar PCNA (3S-binding mode, state 2) at 3.33 Å resolution, a binary complex containing four clamp loader subunits bound to a closed planar PCNA (4S-binding mode, state 3) at 3.16 Å resolution (Fig. 1 *D* and *E*), and four ternary complexes of CTF18-RFC–DNA–PCNA in which all five clamp loader subunits bind PCNA and 3′-ss/ds DNA is located within the CTF18-RFC central chamber and threads through PCNA (5S-binding modes, states 4 to 7) (Fig. 1 *F*–*I*). The four 5S-binding mode ternary complexes are different in terms of their extent of PCNA ring opening and PCNA binding with CTF18-RFC: one complex has a 21-Å opened PCNA spiral (state 4) at 2.93 Å resolution (Fig. 1*F*), two other complexes have a similar conformation but narrower PCNA openings of 12 Å and 5 Å (states 5 and 6) at an overall resolution of 2.75 Å and 2.83 Å, respectively (Fig. 1 *G* and *H*), and one complex in which PCNA is closed, but is nonplanar which we refer to as a “cracked” PCNA interface, at 3.01 Å resolution (Fig. 1*I*).

In the binary CTF18-RFC–PCNA complexes, the A-gate between the A′-domain and the AAA+ module of the CTF18 subunit is closed (Fig. 1 *D* and *E*), and the binary complex becomes more compact transitioning from the 3S-binding mode (state 2) to the 4S-binding mode (state 3). In contrast, the A-gate is open and occupied by the 3′-overhang of the template strand in the CTF18-RFC–PCNA-DNA ternary complexes, and the PCNA ring is open and becomes a right-handed spiral, with a gap size that decreases from 21 Å (state 4) to 12 Å (state 5) to 5 Å (state 6) and 0 Å (state 7) (Fig. 1 *F*–*I*). The 21 Å open gap is wide enough to allow dsDNA to enter PCNA and the three smaller gaps likely represent post-DNA entry intermediates.

Although the intact CTF18-RFC complex was used in this study, the hook module composed of DCC1, CTF8, and the N-terminal peptide of Ctf18 was not visible, likely due to the hook’s long/flexible link to the main body AAA+ module) consistent with a previous study of the yeast Ctf18 hook module (46–48). Importantly, no external shoulder DNA binding was observed in any of the seven EM maps, despite the use of the double-tailed DNA substrate. This result is consistent with previous ensemble studies demonstrating the recognition of only 3′-recessed DNA by CTF18-RFC (26) and suggests that CTF18-RFC does not have a shoulder 5′ DNA site, as further described below.

### The CTF18 Collar Domain and AAA+ Module Have Evolved to Be Mobile.

**Fig. 2.**
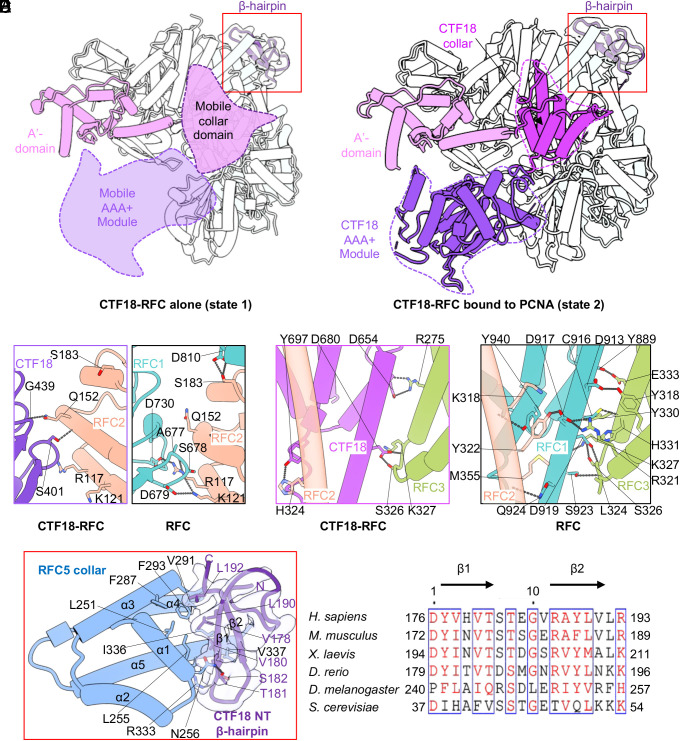
The CTF18 AAA+ module and collar domain are intrinsically mobile and stabilized only when interacting with PCNA. (*A*) Structure of CTF18-RFC showing that both the collar domain and the AAA+ module of CTF18 are mobile in the absence of PCNA (state 1). CTF18 is colored by individual domains, and other subunits are in gray. (*B*) Structure of the CTF18-RFC–PCNA binary complex in the 3S-binding mode, state 2. PCNA is omitted for clarity. The CTF18 AAA+ module and collar domain become stable now by interaction with PCNA. The EM map of the CTF18 N-terminal β hairpin region is superimposed as a transparent light gray surface. (*C*) Comparison of the AAA+ module interfaces between CTF18 and RFC2 and between RFC1 and RFC2 in the human RFC. (*D*) Comparison of the collar domain interfaces among the CTF18, RFC2, and RFC3 in CTF18-RFC and the human RFC. (*E*) Close-up view of the red box region in *A* and *B*, showing the interaction between the RFC5 collar domain and the β hairpin at the CTF18 N-terminal region. Key residues involved in the interaction in *C* to *E* are in sticks and labeled. The EM map of the CTF18 N-terminal β hairpin region is superimposed as a transparent light gray surface. (*F*) Sequence alignment of the β hairpin region of CTF18 from seven eukaryotic organisms.

Surprisingly, there is no density for either the AAA+ module or the collar domain of CTF18 in the apo CTF18-RFC state (Figs. 1*C* and 2*A*), while the densities of the AAA+ module and the collar domain of CTF18 are present in the other 3D maps when PCNA is bound to CTF18-RFC (Figs. 1 *D*–*I* and 2*B*), suggesting that PCNA binding has stabilized the AAA+ module of CTF18. The largest subunit of clamp loaders is the A subunit, which is generally stable and contributes the largest binding interface with PCNA during the clamp opening process of all previously characterized clamp loaders (27–29, 35–38). The uniquely mobile AAA+ module of CTF18 likely slows down the initial docking of CTF18-RFC with PCNA, and this may explain CTF18-RFC’s lower PCNA loading activity compared to RFC (25, 26). Furthermore, the CTF18 collar domain is also mobile in the apo state, leaving only the A′ domain to bind the RFC3 with a small interface, which appears to lack sufficient buried surface to keep CTF18 stably associated with RFC2-5 to form the CTF18-RFC—however, there is, in fact, an additional and unique intersubunit interaction as explained below.

To understand how the CTF18 AAA+ and collar domains are so mobile, yet retain a pentameric structure, we compared its interface with neighboring subunit RFC2 in the S3 binding state 2 structure with that in the human RFC (Fig. 2*C*). We found that RFC1 and RFC2 form two strong salt bridges (Asp-730 with Arg-117 and Asp-697 with Lys-121) in RFC, but the two Asp residues of RFC1 are absent in CTF18, abolishing the two salt bridges in CTF18-RFC, perhaps rendering a very weak interface between CTF18 AAA+ and RFC2 AAA+ modules, and hence the mobile CTF18 AAA+ module as we observe here. In the collar region, the RFC1 collar interacts extensively with both RFC2 and RFC3, involving a network of 12 H-bonds (Fig. 2*D*). In contrast, the CTF18 collar domain only forms 4 H-bonds with RFC3 and no direct interactions with RFC2. This explains the high mobility of the CTF18 collar domain in CTF18-RFC.

### CTF18 Has Evolved a Unique N-terminal β-hairpin to Stabilize the CTF18-RFC Complex.

Interestingly, we found an unexpected density associated with the RFC5 collar domain. Based on the density features and the AlphaFold prediction, this density was identified to be a β-hairpin from the N-terminal region of CTF18 (Fig. 2 *A* and *E*). The β-hairpin inserts into a groove in the RFC5 collar domain lined by helices α1, α3, α5, and the linker loop of α3 and α4 (Fig. 2*E*). The CTF18 β-hairpin residues Val-178, Val-180, Leu-190, and Leu-192 form hydrophobic interactions with the RFC5 Leu-251, Leu-255, Phe-287, Val-291, Phe-293, Ile-336, and Val-337 in the collar domain groove. Thr-181 and Ser-182 of the β-hairpin also form two hydrogen bonds with Asn-256 and Arg-333 of RFC5, respectively. Although CTF18 and RFC5 are separated by RFC2 in the CTF18-RFC complex, the CTF18 subunit projects the N-terminal β-hairpin across RFC2 to directly bind to the RFC5 collar domain. These strong interactions between CTF18 and RFC5 contribute to the CTF18-RFC stability. Sequence alignment among different eukaryotic CTF18’s showed that the β-hairpin is conserved among CTF18 homologs (Fig. 2*F*), underscoring the importance of the β-hairpin region.

### Nucleotide Binding and Progressively Increasing the Interface with PCNA Drives Ring Opening by Ctf18-RFC.

**Fig. 3.**
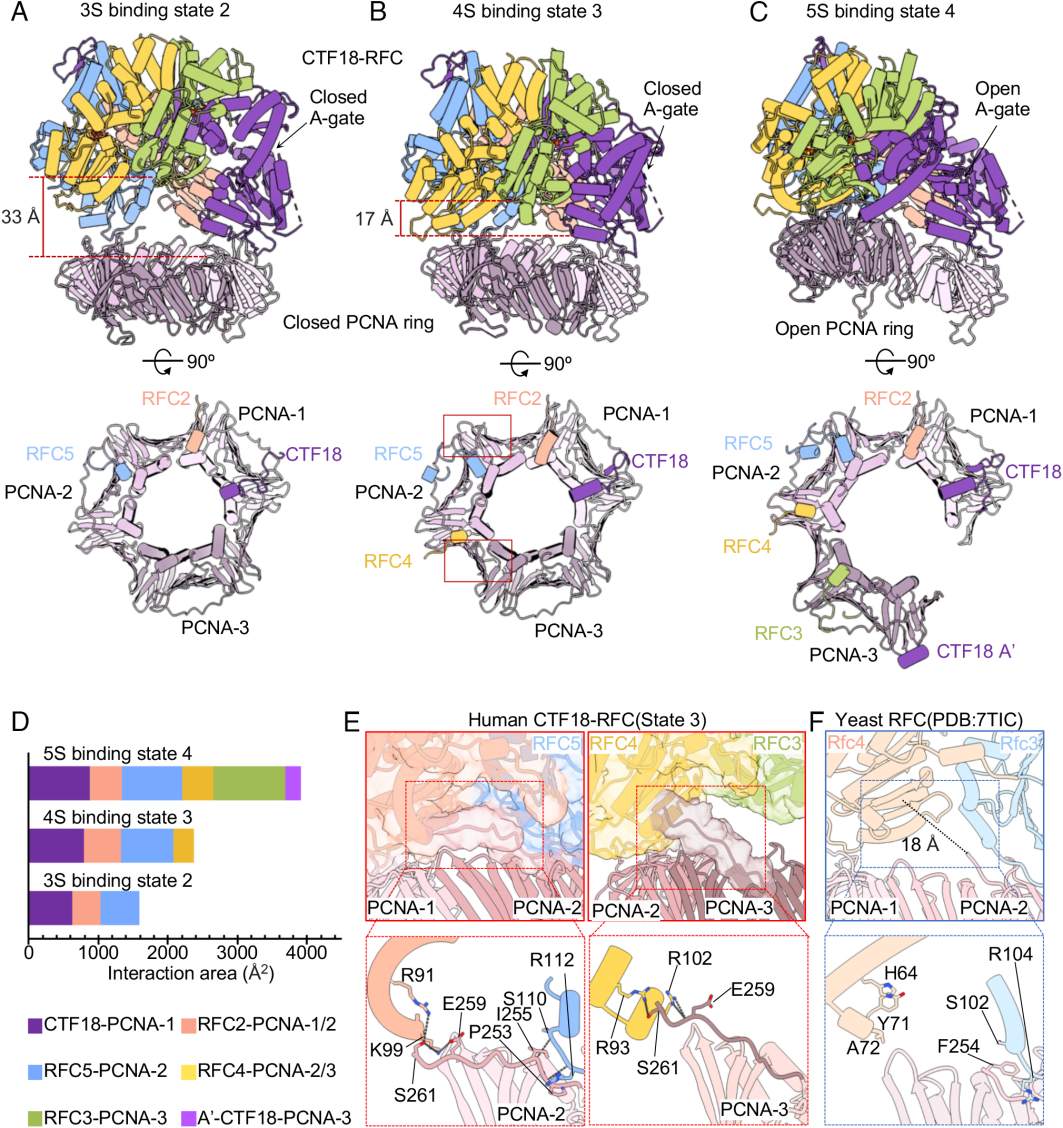
Dynamic interface between clamp and clamp loader in the binary CTF18-RFC–PCNA complex. (*A*–*C*) *Upper* panel: Atomic models of the: (*A*) S3-binding mode, state 2, in which 3-clamp loading subunits-bind PCNA, (*B*) 4S-binding mode, state 3, in which 4 clamp loading subunits bind PCNA, and (*C*) 5S-binding mode, state 4, in which all 5-clamp loading subunits-bind PCNA. These states of CTF18-RFC–PCNA are represented in cartoons and colored by individual subunits. The distance between the bottom of RFC3 and the top of the PCNA ring is labeled in *A* and *B*. The illustrations below show the contacts between CTF18-RFC and PCNA subunits 1–3 viewed from the top. Only PCNA-interacting elements in CTF18-RFC are shown for clarity. (*D*) Plot of the buried surface areas between CTF18-RFC and PCNA in the three states. The contribution of individual subunits is defined by color in each state. (*E*) Close-up views of the two red box regions in *B* showing the EM map transparent surface (*Upper*) and the structures of the C-termini of PCNA-2 and PCNA-3 interacting with the RFC2 (*Left*) and RFC4 AAA+ modules (*Right*), respectively. (*F*) Close-up view of Rfc4, Rfc3, and the C-terminus of PCNA2 from the yeast RFC–PCNA model (PDB entry 7TIC). The last resolved residue at the C-terminus of yeast PCNA is 18 Å away from Rfc4. The corresponding residues in CTF18-RFC and RFC are labeled.

Comparison of all CTF18-RFC–PCNA structures (±DNA) are similar and resemble other RFC–PCNA structures in that they form a three-tiered architecture with the PCNA ring forming the bottom tier, and the five collar domains and five AAA+ modules of CTF18-RFC form the top and middle tiers (Fig. 3 *A*–*C*). The resolution of our cryo-EM maps was sufficient to identify the bound nucleotides (*SI Appendix*, Fig. S4). We identified four ATPγS molecules in the AAA+ nucleotide binding pockets of subunits CTF18/A, RFC2/B, RFC5/C, and RFC4/D, and one ADP in subunit RFC3/E (*SI Appendix*, Fig. S4 *A*–*C*), while the density for ATPγS in CTF18/A of the 3S-binding mode (state 2) and ATPγS in RFC4/D of the 4S-binding mode (state 3) are not clear due to the low local resolution. The overall nucleotide binding pattern in CTF18-RFC is consistent with those observed in other clamp loaders like RFC and RAD17-RFC (yeast Rad24-RFC) (27–29, 35–38). In the 3S-binding mode (state 2), in which 3 clamp loader subunits bind PCNA, the ATPase sites of all the subunits are in an inactive state (*SI Appendix*, Fig. S4*A*) because there is no arginine finger from the adjacent subunit to stabilize the bound ATPγS and the catalytic Mg^2+^ ion. In the 4S-binding mode (state 3), in which 4 clamp loader subunits bind PCNA, only the ATPase site in the CTF18/A subunit is in the active state, the arginine fingers from the adjacent subunits weakly interacted with the bound ATPγS in the B and C subunits, and there is no contact of an arginine finger to ATPγS in the D subunit (*SI Appendix*, Fig. S4*B*). In the 5S-binding modes, in which all five clamp loader subunits engage PCNA, the ATPase sites of A, B, C, and D subunits are all in the active state as the arginine fingers from the adjacent subunits are in contact with the γ-phosphate of the bound ATPγS (*SI Appendix*, Fig. S4*C*).

In the 3S-binding mode (state 2), the AAA+ modules of CTF18, RFC2, and RFC5 interact with PCNA-1 and PCNA-2. CTF18 and RFC5 bind in the main hydrophobic grooves between the N- and C-terminal domains of PCNA-1 and PCNA-2, respectively. RFC2 binds at the intersubunit interface between PCNA-1 and PCNA-2. Transitioning from the 3S- to the 4S-binding modes (states 2 to 3), RFC4 reaches down to bind at the interface between PCNA-2 and PCNA-3, reducing the vertical distance between RFC3 and PCNA from 33 Å to 17 Å (Fig. 3 *A* and *B*). Structural alignment indicates that this transition involves only rigid-body motion of the PCNA ring and CTF18-RFC approaching each other (*SI Appendix*, Fig. S5 *A* and *B*). From the 4S- to the 5S-binding modes (states 3 to 4/5/6/7), RFC3 joins the other four subunits to interact with PCNA and converts the PCNA ring into a right spiral that closely matches the bottom spiral shape of CTF18-RFC (Fig. 3*C*). In state 4 (example of a 5S-binding mode), CTF18/A, RFC5/C, and RFC3/E insert their respective canonical or degenerate PIP motifs into the PIP-binding pockets of PCNA (*SI Appendix*, Fig. S6), and RFC2/B, RFC4/C, and the CTF18 A’-domain form weak interactions with PCNA. The interface between the loader and the clamp increases progressively from ~1,600 Å^2^ [the 3S-binding mode (state 2)] to ~2,400 Å^2^ [the 4S-binding mode (state 3)] and to ~3,900 Å^2^ [the 5S-binding mode (state 4)] (Fig. 3*D*), Therefore, we suggest that the increasing surface area of interaction between Ctf4-RFC-to-PCNA drives PCNA ring opening (*SI Appendix*, Fig. S6 *A*–*C*).

Interestingly, we found in the 4S-binding mode (state 3) structure that the C-termini of PCNA-2 and PCNA-3 interact with RFC2 and RFC4, respectively (Fig. 3 *B* and *E*). Specifically, two positively charged residues in RFC2 (Arg-91 and Lys-99) and one positively charged residue in RFC4 (Arg-93) interact with the C-terminal carboxylate of Ser-261 in PCNA-2 and PCNA-3, respectively (Fig. 3*E*). In the yeast RFC–PCNA complex structure (26–29), the C-terminal residues of the PCNA are disordered and the last resolved residue Phe-254 is 18 Å away from the corresponding interaction region of the RFC4 subunit in CTF18-RFC. Interestingly, the PCNA carboxylate-interacting residues in CTF18-RFC are not conserved in the yeast RFC (Fig. 3*F*). Despite this difference, the overall interactions between CTF18-RFC and PCNA are comparable to those observed in the RFC–PCNA structures (35–38).

### CTF18-RFC Undergoes a Large Conformational Change to Open the PCNA Ring.

**Fig. 4.**
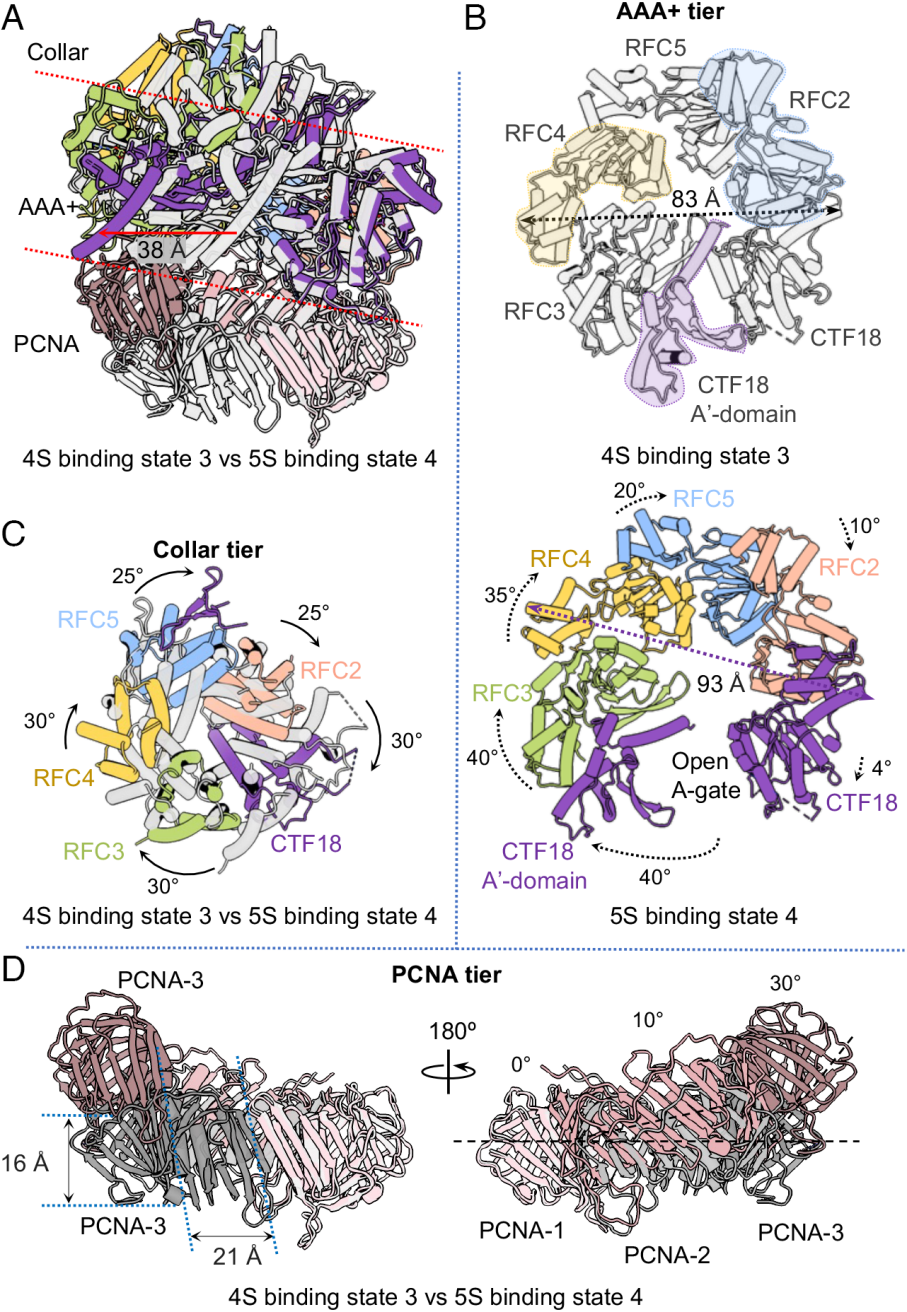
CTF18-RFC undergoes a large conformational change to open the PCNA ring. (*A*) Superposition of the 4S-binding mode (four clamp loader subunits-PCNA) state 3 (gray) and the 5S-binding mode (five clamp loader subunits-PCNA) state 4 (color) of these two binary CTF18-RFC–PCNA complex structures in a side view. Two dashed lines separate the collar tier, the AAA+ tier, and the PCNA tier. (*B*) Top view of the AAA+ tiers of the 4S- and 5S-binding states showing that the CTF18 A′-domain (gray) blocks the access to the central DNA-binding chamber in the 4S-binding mode, state 3, and the CTF18 AAA+ module (purple) rotates 40° and moves 38 Å to open the A-gate for DNA entry in the 5S-binding mode, state 4. The diameter of the AAA+ tier is 83 Å and 93 Å in the 4S- and 5S-binding modes (states 3 and 4, respectively). (*C*) Top view of the superimposed collar tiers of the two states showing a smaller rigid-body rotation in the top collar tier. (*D*) Structural overlay of the PCNA tiers of the two states shows that PCNA-2 and PCNA-3 undergo rigid movements that convert the planar ring to a right-handed spiral. PCNA-3 moves left by 21 Å and up by 16 Å and then tilts by 30°, and PCNA-2 tilts by 10° in place transitioning from the 4S-binding mode, state 3 to the 5S-binding mode, state 4.

From the 3S- to 4S- to 5S-binding modes, CTF18-RFC descends spirally, enabling 3, then 4, then 5 clamp loader subunits to interact with PCNA and crack open the ring (Movie S1). However, the largest conformational changes occur from the 4S- to 5S-binding modes involving 4 and 5 clamp loader subunits bound to PCNA, and changes are observed in all three tiers of the CTF18-RFC–PCNA complex (Fig. 4 *A*–*D*). The AAA+ module of each subunit rotates counterclockwise, with a gradually increasing rotation angle of 4°, 10°, 20°, 35°, and 40° for CTF18/A to RFC2/B, RFC5/C, RFC4/D, and RFC3/E, respectively (Fig. 4*B*). These rotations increase the diameter of the AAA+ tier from 83 Å to 93 Å. Importantly, the CTF18 A′-domain rotates 40° together with the RFC3 AAA+ module, creating a 38-Å gap between the AAA+ module and the A′-domain of CTF18/A, leading to opening the CTF18 A-gate (Fig. 4 *A* and *B*). However, all five collar domains rotate counterclockwise by ~25° to 30° from the 4S- to the 5S-binding modes, rather than moving together with their associated AAA+ modules (Fig. 4*C*). Such structural changes are consistent with the suggested role of the collar tier in holding together the clamp loader complex (50). The structural changes in CTF18-RFC result in PCNA-3 moving 21 Å away from PCNA-2 and 16 Å up, equivalent to a 30° tilt upward by PCNA-3 (Fig. 4*D*). PCNA-2 also tilts slightly up by ~10°, but PCNA-1 is essentially stationary.

### CTF18-RFC Interaction with DNA in the Central Chamber Largely Resembles that of RFC.

**Fig. 5.**
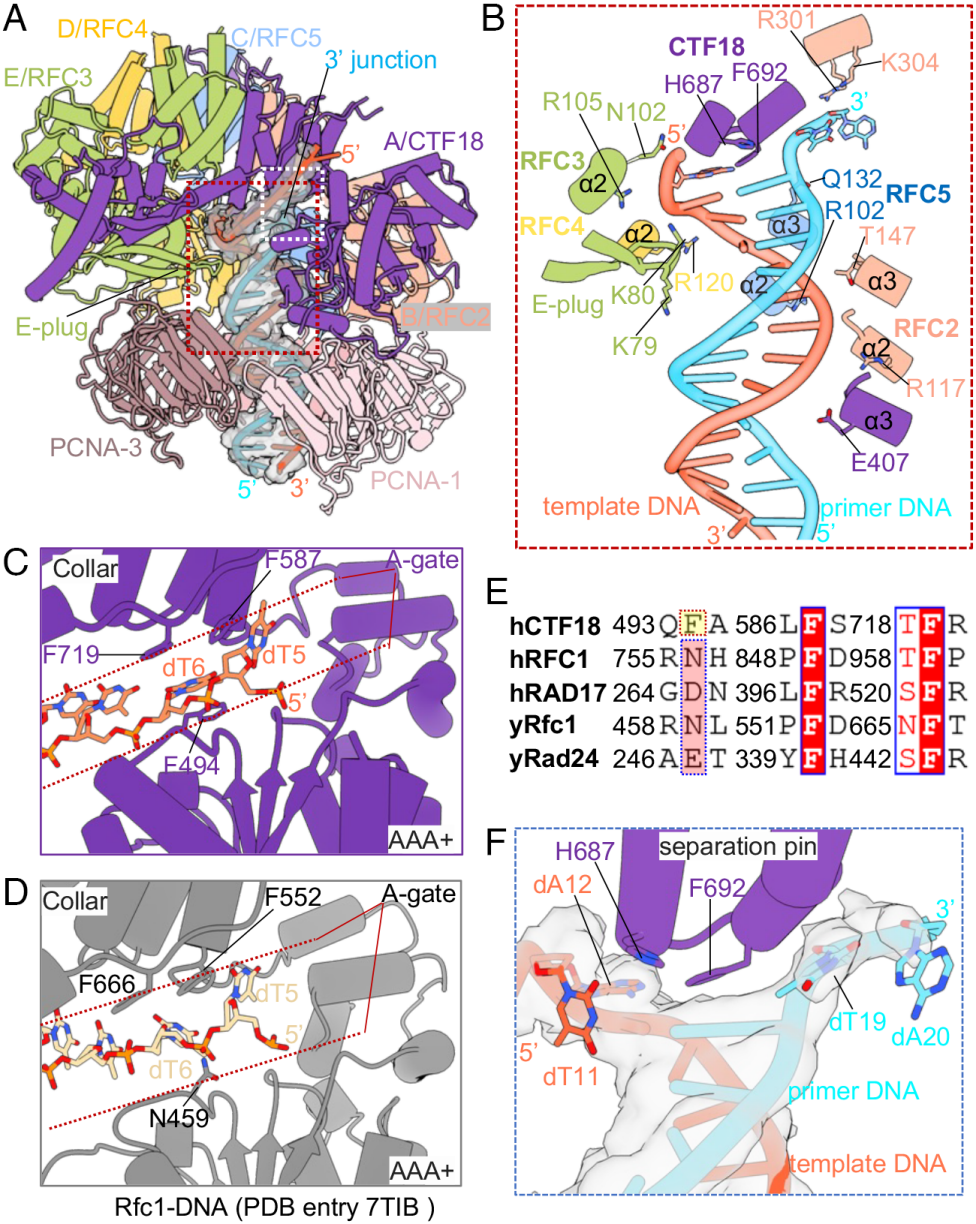
Interactions between CTF18-RFC and the DNA substrate with a 3′-ss/dsDNA junction. (*A*) Structure of the ternary CTF18-RFC–DNA–PCNA complex in state 5 having DNA and all five clamp loading subunits contacting PCNA. The DNA density is shown on a transparent surface and superimposed with the atomic model. The E-plug inserts into the DNA major groove. Regions in the red and white boxes are enlarged in *B* and *C*. (*B*) Detailed interactions between CTF18-RFC and the 3′-ss/dsDNA junction. The melted DNA bases are shown in sticks. α-Helix 3 (α3) of the CTF18 AAA+ module and each of the four α2 helices of the AAA+ modules of RFC2, 5, 4, and 3 wrap around the template strand (firebrick). Helix α3 of the RFC5 AAA+ module interacts with the primer strand. The E-plug residues K79 and K80 H-bond with the primer and template DNA, separately. The DNA-contacting residues are shown as sticks. (*C*) Interaction of the template 5′-overhang in the CTF18 A-gate (red dotted region). Phe-494, Phe-587, and Phe-719 stabilize the template bases. (*D*) Interactions of the template 5′-overhang in the Rfc1 A-gate (red dotted region). Phe-552 and Phe-666 stabilize the template bases. (*E*) Sequence alignment in the template 5′-ssDNA-interacting regions of the human and yeast CTF18, RFC1/Rfc1, RAD17/Rad24. The conserved base-stabilizing Phe residues are in red. (*F*) The separation pin in the CTF18 collar domain melting the 3′-ss/dsDNA junction. The unwound base pairs (dA-12: dT-19 and dT11: dA20) are shown in sticks. The EM density of the bound DNA is shown as a gray surface.

The four CTF18-RFC–DNA–PCNA ternary complex structures (5S-binding modes, states 4 to 7) are similar in the CTF18-RFC region but different in the PCNA region (Fig. 1 *F*–*I*). Here, we choose the state-5 structure with the best resolution (2.75 Å) to describe interactions between the loader and DNA (Fig. 5 *A*–*E*). The 20-bp duplex region of the DNA is fully stabilized by the central chamber of CTF18-RFC and PCNA, and six nucleotides of the 10-nt 5′-ssDNA overhang are stabilized by the open CTF18 A-gate (Fig. 5*A*). Similar to the T4 and yeast RFC clamp loaders (33–38), a series of short α-helices (α2 and α3) in the AAA+ modules of CTF18/A, RFC2/B, RFC5/C, and RFC4/D form a right-hand spiral to bind the template strand and track the minor groove of DNA duplex, and the conserved RFC3/E β-hairpin (the E-plug) inserts into the major groove (Fig. 5*B*). Specifically, CTF18 α3 residues Ser-405 and Glu-407, RFC2 α2 Arg-117 and α3 Thr-147, RFC5 α2 Arg-102, RFC4 α2 Arg-120, and RFC3 α2 Asn-102 and Arg-105 H-bond with the template strand phosphate backbone. Also, the E-plug residue Lys-80 forms an H-bond with the template strand. The primer strand is stabilized by Arg-301 and Lys-304 of the RFC2 collar domain, Gln-132 of RFC5 α3, and the E-plug Lys-79.

The 5′-overhang of the 3′ primed DNA lines the CTF18 A-gate. The dT6 base is sandwiched between the two phenyl rings of the AAA+ module Phe-494 and the collar domain Phe-719, and the dT5 base stacks against the phenyl ring of the collar domain Phe-587 (Fig. 5*C*). In comparison, the yeast RFC A-gate is also lined by the 5′-overhang of the 3′ primed DNA, with two phenyl rings of the Rfc1 collar domain Phe-552 and Phe-666 forming π–π interactions with the bases of dT5 and dT6, respectively (35, 36) (Fig. 5*D*). The first 5′-overhang interacting CTF18 residue (Phe-494) is not conserved, but the other two residues (Phe-587 and Phe-719) are well conserved (Fig. 5*E*). Overall, CTF18-RFC interacts with the chamber DNA in a highly similar fashion to RFC.

### CTF18-RFC Does Not Possess an External (Shoulder) DNA Binding Site.

As stated above, despite the use of the DNA substrate with both 3′- and 5′-overhangs, we only observed DNA in the internal chamber of CTF18-RFC. This is clearly different from the canonical clamp loader RFC as well as the 9-1-1 clamp loader RAD17 (yeast Rad24) which bind both 3’- and 5’-overhangs of two DNA molecules in two distinct sites (27–29, 35–37). To understand the lack of an external shoulder 5′ DNA site in CTF18-RFC, we examined the surface electrostatic charge distribution of the CTF18 AAA+ module and found that the shoulder is highly negatively charged, in contrast to the highly positively charged 5’ DNA binding shoulders of yeast Rfc1 and Rad24, and human RAD17 (*SI Appendix*, Fig. S7*A*). The reversed charge distribution likely explains why the CTF18 shoulder does not bind DNA. Further structural analysis revealed that the P-loop NTPase domain of the CTF18 AAA+ module contains an extra helix (α1) compared to other clamp loaders (*SI Appendix*, Fig. S7*B*), and superimposition of the CTF18 AAA+ module with those of Rfc1, Rad24, and RAD17 demonstrates that the α1-helix and the associated loop in CTF18 sterically clash with the shoulder DNA (*SI Appendix*, Fig. S7*B*). Therefore, CTF18 has reversed the surface charge in the shoulder region and evolved an extra helix to prevent DNA binding at the external shoulder site.

### CTF18 Contains a DNA Separation Pin.

Interestingly, we found that CTF18 also contains a DNA separation pin that was first observed in a collar domain of the bacterial β-clamp loader (34) (Fig. 5*F*). The separation pin residue Phe-692 has disrupted two base pairs from the 3′-end of the primer: The primers dT-19 and dA-20 (Fig. 1*B* and *SI Appendix*, Fig. S1*B*) have been peeled off from the template strand and are flipped out (Fig. 5*F*). The phenol ring of the separation pin residue Phe-692 displaces dT-19 to stack with the template dA-12, and His-687 stabilizes the template dA-12 via a π–π interaction (Fig. 5*F*). Duplex DNA melting at the 3′-ss/ds DNA junction by a separation pin is likely a conserved feature of eukaryotic PCNA loaders because a separation pin has also been observed in RFC (35–37). Comparing the separation pins and the E-plugs of CTF18-RFC and RFC structures, we found that there are variations in the 3′-end base flipping, but the E-plug always inserts into the middle of the major groove of the bound DNA (*SI Appendix*, Fig. S8). Thus, the separation pin in CTF18 or Rfc1 unwinds the base pairs at the 3′-junction DNA so that the E-plug can recognize the middle major groove of the dsDNA, which suggests that both the conserved separation pin and the E-plug work together to “measure the distance” between the clamp loader dsDNA and different 3′-ss/ds DNA junctions in the central chamber.

### PCNA Clamp Closure Is Driven by DNA and Is Independent of ATP Hydrolysis by CTF18-RFC.

**Fig. 6.**
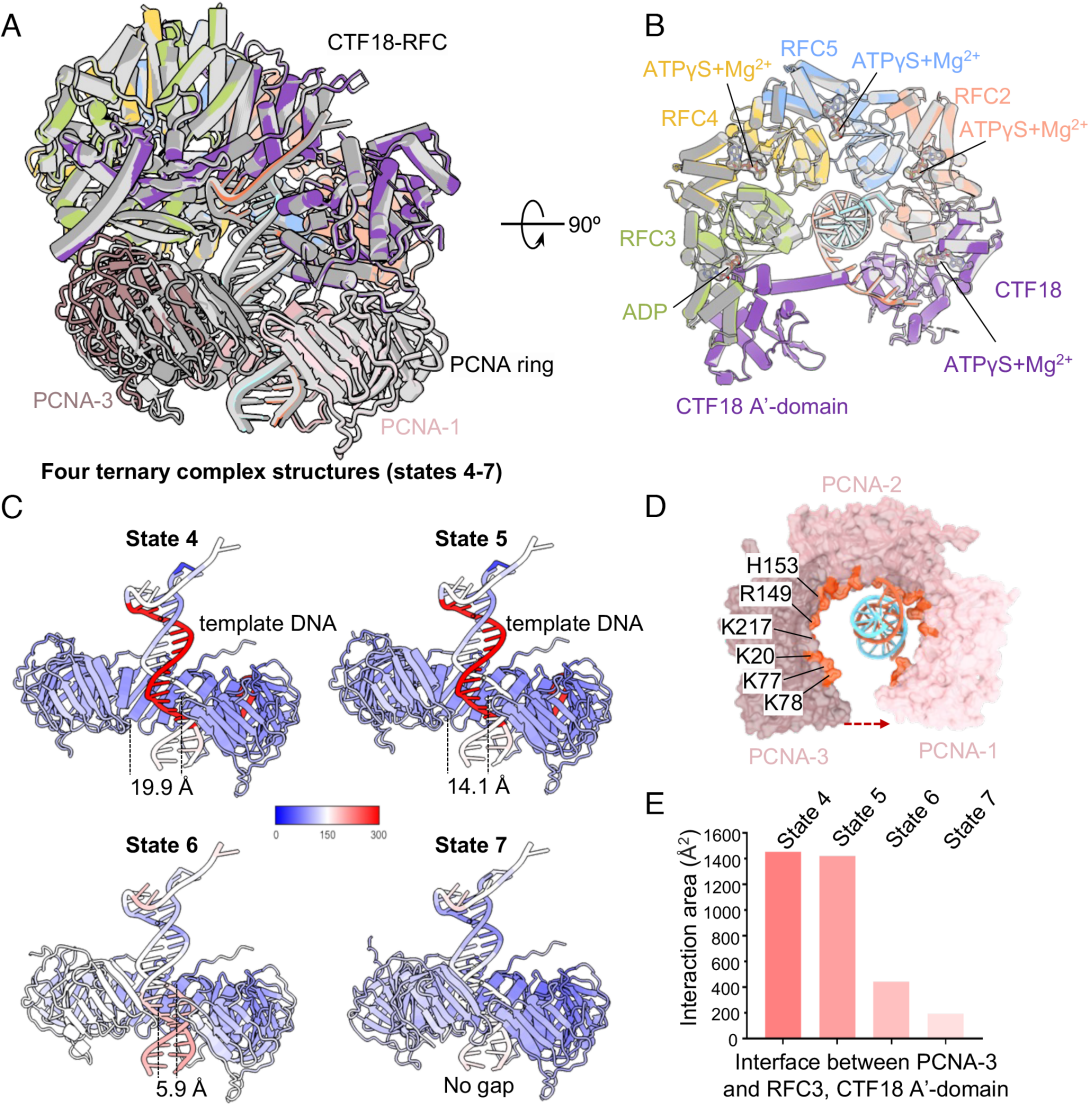
DNA induces PCNA ring closure independently of ATP hydrolysis by CTF18-RFC. (*A*) Superimposition of the four CTF18-RFC–DNA–PCNA ternary complex structures (5S binding modes) derived from state 4 (color), and states 5, 6, and 7 (gray), in which all 5 clamp loader subunits bind the PCNA ring that is open to various extents, revealing large conformational changes in closing of the PCNA clamp, particularly in PCNA-3. (*B*) Top view of the superimposed AAA+ tiers revealing similar conformation and the same nucleotide binding state among these four structures (i.e., states 4 to 7). (*C*) Flexibility analysis of the bound DNA substrates and the PCNA ring in these four structures. The atomic models are colored based on their corresponding local B factors in ChimeraX. The template strands in the first two structures (states 4 and 5) have high B-factors, while PCNA-3 has a high B-factor (flexible) in the third structure (state 6) and a lower B-factor (stable) in the fourth structure (state 7). (*D*) Top view of the PCNA–DNA in the second ternary complex structure (state 5). The PCNA inner surface is lined by positively charged residues. These residues are shown in fire brick and labeled in PCNA-3. (*E*) The contact surface area between PCNA-3 and the loader subunits RFC3 and CTF18 A’-domain in the four ternary complex structures.

Comparison of the four CTF18-RFC–DNA–PCNA 5S-binding mode ternary complex structures reveals that CTF18-RFC is very similar, and conformational changes upon DNA binding are mostly limited to PCNA (Fig. 6*A*). Consistent with the similar CTF18-RFC structures, their nucleotide-binding patterns are the same in the four states (states 4, 5, 6, and 7), with a Mg^2+^-coordinated ATPγS in each nucleotide-binding pocket of CTF18/A, RFC2/B, RFC5/C, and RFC4/D and an ADP in the nucleotide-binding site of RFC3/E (Fig. 6*B*). From state 4 to state 7, PCNA-3 moves progressively closer toward PCNA-1, with the gap size decreasing from 19.9 Å (state 4) to 14.1 Å (state 5), to 5.9 Å (state 6), and 0 Å (state 7), thereby, transforming the PCNA ring from a wide gapped spiral to a closed but nonplanar cracked ring (Fig. 6*C*). Because the CTF18-RFC structure and its associated nucleotides remain the same, the observed conformational changes in the PCNA are not likely to have been caused by ATP hydrolysis by CTF18-RFC.

It is interesting to note that the template strand inside PCNA appears much less stable in states 4 and 5 with larger PCNA gaps than in states 6 and 7 that have smaller or no PCNA gap (Fig. 6*C*). The PCNA clamp is a right-hand spiral in states 4 and 5 in which PCNA-1 and PCNA-2 follow the minor groove of the dsDNA. The PCNA inner face is lined by many positively charged residues such as Lys-20, Lys-77, Lys-78, Arg-149, His-153, and Lys-217 (Fig. 6*D*). It appears that the electrostatic attraction between the negatively charged DNA phosphate backbone and the positively charged inner surface of PCNA drives PCNA ring closure and stabilizes the DNA position in the ring. Accompanying ring closure, PCNA-3 gradually moves down and away from the CTF18 A’-domain and the RFC3 AAA+ module, reducing their binding surface from ~1,400 Å^2^ to ~200 Å^2^ (Fig. 6*E*). Overall, the four ternary complex structures reveal that the charge–charge interactions between the central lumen of PCNA and the dsDNA phosphate backbone drive PCNA ring closure to completely encircle the DNA, and while PCNA is closed but not planar at the last step (state 7), this process is independent of ATP hydrolysis by CTF18-RFC.

## Discussion

The PCNA loading mechanism by the alternative clamp loader CTF18-RFC has been unclear, partly due to the lack of structural knowledge. We have characterized by cryo-EM the in vitro loading mixture of human CTF18-RFC and PCNA with a two-tailed DNA (containing both 3′- and 5′-ss/dsDNA junctions) in the presence of ATPγS. We have determined seven complex structures corresponding to seven PCNA loading intermediate states (states 1 to 7). Morphing these structures provides a nearly complete picture of the PCNA loading cycle by CTF18-RFC (Movie S1).

The study mainly shows two unique features. First, that CTF18 contains a unique mobile CTF18 AAA+ module (compared to other clamp loaders). Second, an external 5′-DNA binding site in CTF18-RFC is not observed, unlike RFC and RAD17-RFC. These features will be discussed below. But for perspective, our study has revealed very large similarities between CTF18-RFC and canonical RFC in loading PCNA onto a 3′-ss/dsDNA junction. Key similarities include the following: i) PCNA loading does not need ATP hydrolysis, and ATP hydrolysis is expected for loader ejection from the loaded PCNA allowing it to form a closed planar PCNA ring around DNA (51–54), ii) The AAA+ module of the first four subunits (A-D) all bind an ATPγS, and only subunit E binds an ADP. iii) The presence of a conserved separation pin that unwinds the duplex from the 3′-end of the ss/dsDNA junction inside the loader ATPase chamber (35–38), iv) the presence of a conserved E-plug that invariantly inserts in the middle of the duplex major groove, and v) the separation pin that likely coordinates with the E-plug to enable the loaders to load PCNA onto any DNA substrate with a 3′-ss/dsDNA junction. Despite these similarities, two important distinctions exist, specifically the unexpected mobile CTF18 AAA+ module and collar domains, and the absence of an external shoulder 5′ DNA binding site (further discussed below).

### A Nearly Complete PCNA Loading Cycle.

**Fig. 7.**
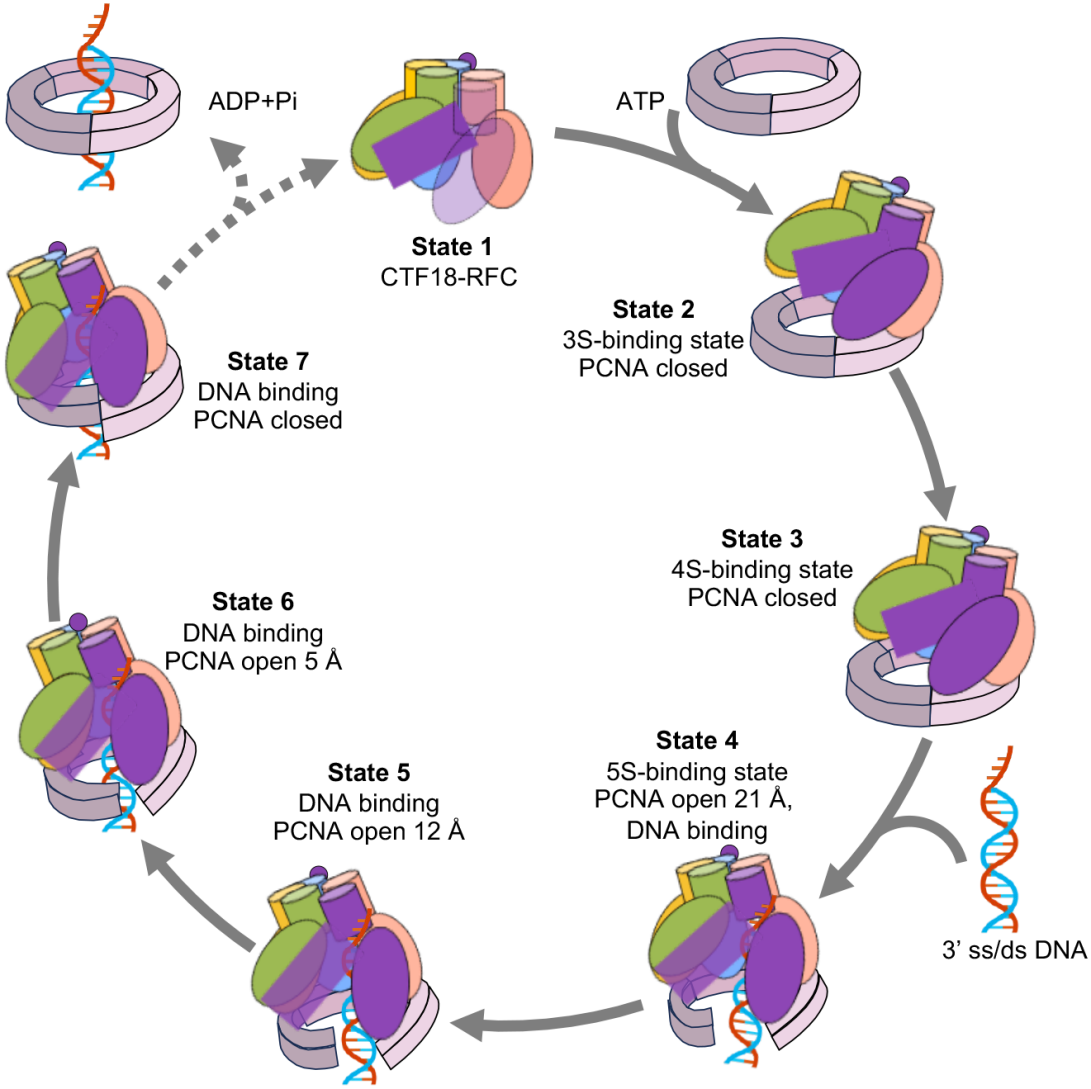
A detailed process of PCNA loading onto the 3′-ss/ds junction DNA by the human CTF18-RFC. In state 1 before engaging a PCNA clamp, the CTF18 AAA+ module and the collar domain are flexible in the ATPγS-bound CTF18-RFC. In state 2 (3S-binding mode), the initial binding of CTF18-RFC to a closed PCNA ring involves three loader subunits (CTF18/A, RFC2/B, and RFC5/C), and the binding stabilizes the otherwise flexible CTF18 AAA+ module and collar domain. In state 3 (4S-binding mode), one more loader subunit (RFC4/D) joins the interaction with the clamp, which is still insufficient to open the A-gate in the clamp loader. In state 4 (5S-binding mode), the last subunit RFC3/E joins the clamp interaction to significantly increase the contact interface, leading to the RFC A-gate opening, PCNA ring opening, and a concomitant entry/binding of the 3′-ss/ds junction entering into the clamp loader and the open PCNA ring. The dsDNA region inside the loader central chamber is stabilized by the E-plug engaging the DNA major groove. CTF18 contains a pair of aromatic residues that function as the separation pin to melt the DNA from the 3’-end of the primer. From states 4 to 6, the encircled DNA induces conformational changes in the CTF18-RFC–PCNA complex in an ATP-hydrolysis independent manner, leading to gradual PCNA ring closure. Finally, in state 7, the closed PCNA ring likely stimulates ATP hydrolysis by CTF189-RFC, leading to the ejection of CTF18-RFC from the DNA-encircling clamp.

The seven distinct structures observed herein extend the major steps of PCNA loading by a clamp loader (Fig. 7 and Movie S1). State 1 represents CTF18-RFC before it encounters PCNA. States 2 and 3 represent binary encounters between the loader and the clamp, with the 3S-binding mode (state 2) and the 4S-binding mode (state 3), that differ by having 3 versus 4 clamp loader subunits that bind PCNA (Figs. 1 *D* and *E* and 3 *A* and *B*). Interestingly, the transition from 3 to 4 clamp loader subunits binding the clamp (3S- to 4S-binding mode, states 2 to 3) involve only rigid-body movements of the CTF18-RFC and PCNA approaching each other (*SI Appendix*, Fig. S5). The 5S-binding mode (state 4) is a key intermediate in which all five clamp loader subunits engage PCNA and drive PCNA to open 21 Å, sufficient to admit dsDNA into PCNA (and into the clamp loader). States 5 and 6 represent post-DNA-entry intermediates in which the bound DNA drives the gradual narrowing of the PCNA gap to 12 Å and 5 Å, respectively, apparently via electrostatic attraction of the positively charged inner surface of PCNA to the negatively charged DNA phosphate backbones. State 7 represents the near-final step of PCNA loading by CTF18-RFC in which the PCNA gap is completely closed, but PCNA-3 and PCNA-1 are still staggered such that the PCNA is still spiral and has yet to convert to a planar ring. In a subsequent state not observed in the current study, we suggest that CTF18-RFC hydrolyzes ATP to disengage from PCNA, leaving the PCNA to spontaneously form a planar ring encircling DNA. However, it remains possible that ring planarity is driven by ATP hydrolysis in CTF18-RFC.

Several recent cryo-EM studies have captured the 3S- and 5S-binding modes but not the 4S-binding mode in the yeast RFC–PCNA complex (35–38). Moreover, the 5S-binding mode, with all 5 RFC subunits bound to PCNA, was captured only in the absence of DNA in a chemically cross-linked RFC–PCNA sample (35). We suggest that CTF18-RFC alone has a lower PCNA loading activity than RFC, consistent with previous ensemble assays (25) and that the low activity may have allowed us to capture the unique number of PCNA loading intermediates of the CTF18-RFC. Consistent with this suggestion, the PCNA loading activity of CTF18-RFC was previously shown to be stimulated by Polε, an essential partner of CTF18-RFC for PCNA loading onto the leading strand DNA (26, 48).

### Potential Functions of the Mobile Collar and AAA+ Module of CTF18.

The low PCNA loading activity is likely an intrinsic property of CTF18-RFC, conferred by the high mobility of the AAA+ module and the collar domain of CTF18. Unique to the Ctf18-RFC clamp loader, both the collar and AAA+ module are flexible before CTF18-RFC encounters the closed PCNA (Fig. 1*B*). The A subunit of clamp loaders (also the largest), such as Rfc1 of RFC and Rad24 of Rad24-RFC, provide the largest contact surfaces with their target clamps to drive clamp opening/loading and appear highly stable in RFC and Rad24-RFC (27–29, 35–37). It is likely that a stable and preformed binding surface is more efficient for interaction with the clamp than a flexible and yet-to-be-formed surface. We propose that CTF18 has evolved a largely mobile architecture to lower its PCNA loading activity, possibly to target loading activity to the leading strand Pol ε to which it is tethered and/or to avoid competition with the canonical RFC clamp loader. However, the mobile architecture of CTF18 can lead to two adverse consequences: 1) an unstable CTF18-RFC complex and 2) the associated low efficiency in loading PCNA to leading strand DNA. The first issue is addressed by evolving an additional binding module—the CTF18-specific N-terminal β-hairpin motif, which has a uniquely significant interaction with RFC5. This interaction, together with the CTF18 A′-domain binding to RFC3, is apparently sufficient to stabilize the CTF18-RFC complex. The second issue is likely addressed by evolving additional subunits DCC1 and CTF8, enabling the loader to interact with Polε, increasing the local concentration of Ctf18-RFC at sites of leading strand synthesis.

### Why Has CTF18-RFC Evolved to Eliminate the External/Shoulder DNA Binding Site?

Our structural analysis revealed that the external DNA site shoulder region of CTF18 is negatively charged, incompatible with binding negatively charged dsDNA. Further, the CTF18 AAA+ module has evolved an extra α1 helix that blocks DNA binding at a potential external (shoulder) DNA site (*SI Appendix*, Fig. S7). In fact, only the internal chamber of CTF18-RFC that recognizes the 3′-ss/dsDNA junction is occupied by DNA in all seven structures, despite our use of the double-tailed DNA substrate that contains both 3′- and 5′-ss/dsDNA junctions. Our result is consistent with biochemical assays indicating that CTF18-RFC only loads PCNA onto a 3′-ss/dsDNA junction (25, 26).

Rad24-RFC and RFC have been shown to bind DNA at both the internal chamber and external (shoulder) sites (27–29, 35–37). This capability has presumably evolved for the function of these loaders to load their respective PCNA and 9-1-1 clamps onto gapped DNA regions where both 3′- and 5′-ss/dsDNA junctions are present as expected to occur at a template lesion and thus for DNA repair and signaling the DNA damage response. We speculate that CTF18-RFC may simply not have use of a 5′ ss/ds DNA site or may have evolved to eliminate the 5′-ss/dsDNA shoulder DNA site to avoid competing with RFC for loading the PCNA clamp onto gaps. Moreover, it is known that CTF18-RFC forms a stable complex with Polε, while the active site of Polε binds to the 3′-ss/dsDNA. This suggests that CTF18-RFC may exchange with Polε to recognize the 3′ ss/ds DNA for PCNA loading. Both the structural features and the specific Polε binding partner likely underlie how CTF18-RFC loads the PCNA clamp onto the 3′-ss/dsDNA for leading strand synthesis (40).

In summary, our study has revealed a nearly complete PCNA loading cycle of the CTF18-RFC. Further studies are needed to understand how the ATP hydrolysis in CTF18-RFC drives the final ejection of the clamp loader from the DNA-loaded clamp and how CTF18-RFC collaborates with Polε to load PCNA onto the leading strand DNA.

## Method Summary

Human CTF18-RFC was expressed and purified using the Bac-to-Bac Baculovirus expression system (Thermo Fisher Scientific). Human PCNA was expressed and purified from *E*scherichia* coli* BL21. In vitro assembly of the human CTF18-RFC–DNA–PCNA complex for cryo-EM used a two-tailed DNA substrate with a 10-nt 3′-recessed end and a 10-nt 5′-recessed end as in our previous study of RFC (37). Reactions contained 1.0 μM CTF18-RFC, 3 μM PCNA, and 10 μM DNA substrate in 20 μL reaction buffer (40 mM HEPES pH 7.5, 0.5 mM TCEP, 1 mM MgAc, 0.5 mM ATPγS, and 40 mM potassium glutamate). The final molar ratio of CTF18-RFC: PCNA: two-tailed DNA was 1:3:10. The reaction mixture was incubated at room temperature for 30 min. Cryo-EM grids were prepared in an FEI Vitrobot Mark IV using holey carbon grids (Quantifoil Au R2/1, 400 gold mesh). Cryo-EM datasets were collected on a 300 kV Titan Krios electron microscope at a scope magnification of 105,000× and an objective lens defocus range of −1.2 to −1.8 µm using a Gatan K3 direct electron detector with a total electron dose of 60 e^−^/Å^2^. A total of 18,239 raw movie micrographs were collected for 3D reconstruction. Please refer to *SI Appendix* for detailed Experimental Procedures and 8 supplemental figures.

## Supplementary Material

Appendix 01 (PDF)

Movie S1. **Overview of human CTF18-RFC action in loading of PCNA**. The movie is generated by morphing the seven experimental states, perhaps representing the most realistic action sequence of any clamp loader characterized so far. Each of the 5 AAA+ subunits of the CTF18-RFC, and each PCNA monomer, are color-coded according to the legend in the movie. The movie starts with a 360o rotation of the loader in the absence of DNA, then the movements of the loader and opening of PCNA induced by ATPγS binding that provide the open A-gate and PCNA for entry of the 3’ primer-template followed by steps in closing of the PCNA clamp around DNA.

## Data Availability

CryoEM structures data have been deposited in EMBD and PDB (EMD-42406 (55), EMD-42383 (56), EMD-42384 (57), EMD-42386 (58), EMD-42385 (59), EMD-42388 (60), EMD-42389 (61), 8UNJ (62), 8UMT (63), 8UMU (64), 8UMW (65), 8UMV (66), 8UMY (67), and 8UN0 (68)).
