# Supplementary material for: Cryo-EM reveals a nearly complete PCNA loading process and unique features of the human alternative clamp loader CTF18-RFC
Source: Proc Natl Acad Sci U S A. 2024 Apr 26;121(18):e2319727121. doi: 10.1073/pnas.2319727121 (PMC11067034; doi:10.1073/pnas.2319727121)
Supplement: Supplementary file 1 — Appendix 01 (PDF) [file pnas.2319727121.sapp.pdf]

Supplementary Information Appendix for:

Cryo-EM reveals a nearly complete PCNA loading process and unique features of the human alternative clamp loader CTF18-RFC

Qing He [1,3], Feng Wang [1,3], Michael E. O'Donnell [2,*], and Huilin Li [1,*]

[1] Department of Structural Biology, Van Andel Institute, Grand Rapids, Michigan, USA

[2] DNA Replication Laboratory and Howard Hughes Medical Institute, The Rockefeller University, New York, New York, USA

[3] These authors contributed equally to this work.

[*] Correspondence should be addressed to M.E.O. (odonnel@rockefeller.edu) or H.L. (Huilin.Li@vai.org)

This Appendix includes:

- **Detailed Experimental Procedures**
- **1 Table**
- **1 Supplementary Video legend**
- **8 Supplementary Figures**
- **References for Supplementary Material**

MATERIALS AND METHODS

Generation of DNA constructs. The *Homo sapiens* cDNAs encoding the proteins studied in this report were purchased from the DNASU Plasmid Repository. The human CTF18 cDNA with an N-terminal 3xFLAG tag was cloned into the pFast-Bac vector (Thermo Fisher). The N-terminal 6xHis-tagged human CTF8 and DCC1 were also cloned into the pFast-Bac-Dual vector. The cDNAs of human RFC2, RFC3, RFC4, and RFC5 were cloned into the pFL multi-gene expression acceptor vector (pLIB_RFC2-5) following the biGBac protocol with minor modifications (1). The human PCNA with an N-terminal His tag followed by a thrombin cleavage site was cloned into the pET28a vector. All constructs were sequenced to verify that no mutations were introduced during PCR and cloning.

Protein expression and purification. The CTF18-RFC complex was expressed by using the Bac-to-Bac Baculovirus expression system (Thermo Fisher Scientific). Three baculoviruses encoding CTF18, CTF8 plus DCC1, and the RFC2-5 subcomplex were used to co-infect Sf9 cells or Hi5 cells (5×10^5 cells per ml) followed by incubation for 72 h at 27°C with constant shaking (115 rpm). To purify CTF18-RFC, harvested insect cells were lysed in buffer (25 mM HEPES, PH7.5, 250 mM NaCl, 1mM Mg-acetate (MgAc), 1 tablet the complete™, EDTA-free protease inhibitors cocktail (Roche Molecular Biochemicals)) by sonication, the lysate was centrifugated in a Ti-45 rotor at 40,000x rpm for 1 h, and the resulting supernatant was incubated with 0.8 mL FLAG-antibody-coated beads at 4°C for 2-3 h. The beads were washed using 100 mL of lysis buffer, and the proteins were eluted with 8 mL of lysis buffer containing 0.2 mg/mL 3 × FLAG peptide. The eluted proteins were concentrated using centrifugal concentrators (Amicon, 100 kDa) and further purified by size exclusion chromatography (SEC, Superose 6 Increase, GE Healthcare) in buffer containing 25 mM HEPES, pH 7.5, 200 mM NaCl, 1mM MgAc, and 1mM DTT. The purified protein was concentrated to 3.2 mg/ml and stored at –80°C.

The human PCNA was expressed and purified from *E. coil* BL21. To produce the PCNA protein in *E. coil* cells, the transformants were grown at 37ºC until the cell density reached an OD_{600} value of 0.8, then 0.2 mM isopropyl-β-D-thiogalactopyranoside (IPTG) was added to induce PCNA expression, and the culture was incubated a

further 12 h at 16°C. Cells were collected, resuspended in buffer A (25 mM HEPES, pH 7.5, 200 mM NaCl, 1 mM MgAc), and lysed with a homogenizer (SPX Corporation). The lysate was centrifuged at 17,000x rpm for 1 h, and the supernatant was loaded into a 5-ml Ni-NTA column (Cytiva). The proteins were eluted using buffer A plus 300 mM imidazole. The N-terminal 6xHis-tag on hPCNA was cleaved by incubating with thrombin at 4°C overnight. The tag-cleaved hPCNA was polished by size exclusion chromatography through a Superdex 200 column (GE Healthcare) in buffer containing 20 mM HEPES, pH 7.5, 200 mM NaCl, 1 mM MgAc, and 1 mM DTT.

In vitro assembly of the human CTF18-RFC–DNA–PCNA complex for cryo-EM. The two-tailed DNA substrate with a 10-nt 3′-recessed end and a 10-nt 5′-recessed end was the same as in our previous study of RFC (37). The 20-nt primer strand was 5′- GCA GAC ACT ACG AGT ACA TA -3′, and the 40-nt template strand was 5′- TTT TTT TTT TTA TGT ACT CGT AGT GTC TGC TTT TTT TTT T -3′. The DNA oligos were synthesized by Eurofins Genomics. The two DNA oligos were annealed by mixing to a final concentration of 66 μM in annealing buffer (20 mM HEPES, pH 7.5, 50 mM NaCl, and 0.5 mM EDTA), heat denatured at 95°C for 10 min, and annealed by gradually decreasing the temperature from 95°C to room temperature. To assemble the human CTF18-RFC-PCNA-DNA complex in vitro, 1.0 μM CTF18-RFC, 3 μM PCNA, and 10 μM DNA substrate were mixed in 20-μl reaction buffer (40 mM HEPES pH 7.5, 0.5 mM TCEP, 1 mM MgAc, 0.5 mM ATPγS, and 40 mM potassium glutamate). The final molar ratio of CTF18-RFC: PCNA: two-tailed DNA was 1:3:10. The reaction mixture was incubated at room temperature for 0.5 h and then stored on ice for 0.5 h until use.

Cryo-EM grids preparation and data collection. Holey carbon grids (Quantifoil Au R2/1, 400 gold mesh) were freshly glow discharged in an Ar/O2 mixture for 30 seconds using a Gatan 950 Solarus plasma cleaning system set at 15 W. Aliquots of 3 μl of the prepared CTF18-RFC-PCNA-DNA loading mixture were applied to the treated EM grids. Following each sample application, the grids were blotted for 3 seconds with a blotting force set to 3, and then rapidly frozen in liquid ethane using an FEI Vitrobot Mark IV. The experiment was conducted with the temperature and relative humidity controlled at 6°C and 100%, respectively. The quality of the EM grids was screened in a 200 kV Arctica cryo-EM equipped with a Gatan K2 direct electron detector. The final high-resolution cryo-EM dataset was collected from high-quality EM grids automatically in the multi-hole mode on a 300 kV Titian Krios electron microscope controlled by SerialEM (2). Micrographs were recorded at a scope magnification of 105,000× with an objective lens defocus range of −1.2 to −1.6 μm using a Gatan K3 direct electron detector operating in the super-resolution video mode, corresponding to the super-resolution pixel size of 0.414 Å. During a 1.0 s exposure time, a total of 50 frames were recorded, with a total electron dose of 60 $e^-/Å^2$.

Image processing and 3D reconstruction. In total, 18,239 raw movie micrographs were acquired and subjected to motion correction using the program MotionCorr 2.0 (3) with 2× binning, resulting in a pixel size of 0.828 Å/pixel. Subsequently, these micrographs were imported into cryoSPARC (4) (version 4.2.1) for patch-based contrast transfer function (CTF) estimation and correction. A total of 16,926 micrographs exhibiting CTF signal extending to 4.0 Å were retained for subsequent processing (SI Appendix, Fig. S2). Blob-based auto-picking (70–130 Å diameter) in cryoSPARC was implemented to select initial particle images and generate 2D templates for subsequent template-based particle picking. A total of 17,504,448 raw particles were automatically picked and extracted. To refine the particle dataset, multiple rounds of 2D classifications were performed on 4x binned particle images, and particles demonstrating clear structural features were chosen for further analysis.

A total of 2,201,365 raw particles extracted with a box size of 320 pixels were used to calculate six starting 3D models. Two 3D reconstructions were discarded as junk particles. Four reliable 3D reconstructions were chosen to perform 3D refinement. Particles in the first 3D class were used to perform a new round of 2D classification, followed by one round of heterogenous 3D refinement to remove bad particles. Sequential homogeneous and non-uniform refinements were employed, resulting in a final 3D map from the first class at an overall resolution of 3.35 Å. The second class was subjected to homogeneous and non-uniform 3D refinements, leading to a final 3D map at an overall resolution of 3.33 Å. The third 3D class was subjected to a heterogeneous 3D refinement to remove bad particles, followed by the homogeneous and non-uniform 3D refinements that resulted in a final 3D map at an overall resolution of 3.16 Å.

The fourth 3D class contained CTF18-RFC bound to both DNA and PCNA. Homogenous 3D refinement led to a 2.57-Å 3D EM map in which the PCNA density was very weak, indicative of the presence of multiple PCNA conformations in this class. Therefore, another round of 3D classification was performed resulting in ten 3D

subclasses. Among the ten subclasses of the CTF18-RFC–DNA–PCNA ternary complex, the PCNA ring in one subclass was in an open spiral conformation. This subclass contained 76,539 particles and was further refined to a final 3D map (the 5S-binding state) at 2.93 Å average resolution. The PCNA ring in three subclasses was an open ring with a 12-Å gap. The particles from these three subclasses were pooled into a consolidated dataset comprising 212,091 particles. Subsequent homogeneous and non-uniform 3D refinements resulted in a 3D map with an average resolution of 2.75 Å (CTF18-RFC–DNA–open PCNA state I). The PCNA in another two subclasses was also an open ring but with a very narrow 5-Å gap. The particles from these two subclasses, totaling 146,250, were combined into a dataset and further refined to produce a final 3D map with an average resolution of 2.83 Å (CTF18-RFC–DNA–open PCNA state II). The PCNA in the remaining subclass was cracked and closed but non-planar. This subclass contained 75,789 particles. Further non-uniform 3D refinement of this dataset resulted in a final 3D EM map at 3.01 Å average resolution (CTF18-RFC–DNA–cracked PCNA).

Model building, refinement, and validation. The AlphaFold2 predicted structures of human CTF18, RFC2-5, and human PCNA were manually docked into the EM maps of the CTF18-RFC alone, and the binary CTF18-RFC–PCNA complex (4S-binding, state 3) in UCSF ChimeraX (5). The models were manually adjusted and rebuilt to fit the EM map in Coot (6). The DNA model was extracted from the yeast RFC–DNA–PCNA complex (37) (PDB ID 7TFI) and fitted into the 2.57-Å intermediate map with high-resolution features in the CTF18-RFC–DNA region but with weak density in the PCNA region. The missing nucleotides were built based on the unsharpened cryo-EM maps. The flipped bases proximal to the separation pin were manually built with Coot. The EM density features allowed unambiguous assignment of the DNA sequence at the 3'-ss/dsDNA junction inside the CTF18-RFC chamber. Moreover, we used the model of a single PCNA subunit to fit the four EM maps of the CTF18-RFC–DNA–PCNA ternary complex. The manually built models underwent multiple rounds of real-space refinement in PHENIX (7) and were manually adjusted in Coot (6). Finally, validation of all atomic models was conducted using MolProbity (8). The EM maps were sharpened by deepEMhancer (9) for refinement of the atomic models and for preparing figures. The EM 3D reconstruction and model refinement statistics are listed in SI Appendix Table S1. Structural figures were generated in the UCSF ChimeraX (5).

Data Availability. The seven EM maps of the human CTF18-RFC alone or bound to PCNA ring have been deposited in the EMDB under accession code EMD-42406 (CTF18-RFC alone, 3.35 Å), EMD-42383 (CTF18-RFC–PCNA, 3.33 Å), EMD-42384 (CTF18-RFC–PCNA, 3.16 Å), EMD-42386 (CTF18-RFC–PCNA, 2.93 Å), EMD-42385 (CTF18-RFC–open PCNA, 2.75 Å), EMD-42388 (CTF18-RFC–open PCNA, 2.83 Å), and EMD-42389 (CTF18-RFC–cracked PCNA, 3.01 Å), respectively. The corresponding atomic models have been deposited in the Protein Data Bank under accession codes 8UNJ, 8UMT, 8UMU, 8UMW, 8UMV, 8UMY, and 8UN0.

SI Appendix, Video S1. Overview of human CTF18-RFC action in loading of PCNA. The movie is generated by morphing the seven experimental states, perhaps representing the most realistic action sequence of any clamp loader characterized so far. Each of the 5 AAA+ subunits of the CTF18-RFC, and each PCNA monomer, are color-coded according to the legend in the movie. The movie starts with a 360o rotation of the loader in the absence of DNA, then the movements of the loader and opening of PCNA induced by ATPγS binding that provide the open A-gate and PCNA for entry of the 3' primer-template followed by steps in closing of the PCNA clamp around DNA.

SI Appendix, Table S1. Cryo-EM data collection, refinement and validation statistics

	CTF18-RFC alone in the apo state (state 1) (EMD-42406) (PDB 8UNJ)	CTF18-RFC-PCNA binary complex in the three-subunit binding state 2 (EMD-42383) (PDB 8UMT)	CTF18-RFC-PCNA binary complex in the four-subunit binding state 3 (EMD-42384) (PDB 8UMU)	CTF18-RFC-PCNA-DNA ternary comp lex in the five-subunit binding state 4 (EMD-42386) (PDB 8UMW)	CTF18-RFC-PCNA-DNA ternary complex with narrow PCNA opening state I (state 5) (EMD-42385) (PDB 8UWV)	CTF18-RFC-PCNA-DNA ternary complex with narrow PCNA opening state II (state 6) (EMD-42388) (PDB 8UMY)	CTF18-RFC-PCNA-DNA ternary complex with closed PCNA (state 7) (EMD-42389) (PDB 8UN0)
**Data collection and processing**							
Microscope				FEI Titan Krios			
Magnification				105,000			
Voltage (kV)				300			
Electron exposure (e–/Å^2)				60			
Defocus range (μm)				–1.2 to –1.6			
Pixel size (Å/pixel)				0.828			
Symmetry imposed				C1			
Initial particle images (no.)				2,201,365			
Final particle images (no.)	90,327	232,472	183,349	76,539	212,091	146,250	75,789
Map resolution (Å)	3.35	3.33	3.16	2.93	2.75	2.83	3.01
FSC threshold	0.143	0.143	0.143	0.143	0.143	0.143	0.143
Map resolution range (Å)	3.0-12.0	2.8-8.4	2.7-6.7	2.5-5.8	2.3-5.6	2.4-5.9	2.6-6.6
**Refinement**							
Model resolution (Å)	4.3	3.7	3.6	3.2	2.9	3.0	3.3
FSC threshold	0.5	0.5	0.5	0.5	0.5	0.5	0.5
Model resolution range (Å)	1.8-5.3						
Map sharpening *B* factor (Å^2)	-106.3	-125.0	-113.4	-92.5	-97.3	-94.5	-90.2
Model composition	3.0-12.0	2.8-8.4	2.7-6.7	2.5-5.8	2.3-5.6	2.4-5.9	2.6-6.6
Non-hydrogen atoms	11916	20697	20541	21860	21860	21860	21860
Protein/DNA residues	1493/0	2625/0	2606/0	2647/47	2647/47	2647/47	2647/47
Ligands	7	7	7	9	9	9	9
B factors (Å^2)							
Protein/DNA	72.78/0	66.55/0	88.91/0	92.33/900	90.21/900	81.48/157	57.55/134
Ligand	72.37	54.57	76.23	54.58	46.85	55.43	43.43
R.m.s. deviations							
Bond lengths (Å)	0.003	0.004	0.006	0.004	0.003	0.004	0.004
Bond angles (°)	0.673	0.750	1.015	0.687	0.684	0.991	0.802
Validation							
MolProbity score	2.07	2.20	1.61	1.67	1.62	1.66	1.53
Clashscore	6.81	9.07	8.54	6.48	7.07	6.09	6.27
Poor rotamers (%)	5.88	4.14	0.84	1.90	1.69	2.34	0.56
Ramachandran plot							
Favored (%)	97.43	96.35	97.21	97.52	97.75	97.79	96.87
Allowed (%)	2.57	3.61	2.79	2.48	2.25	2.21	3.09
Disallowed (%)	0.00	0.04	0	0	0	0	0.04

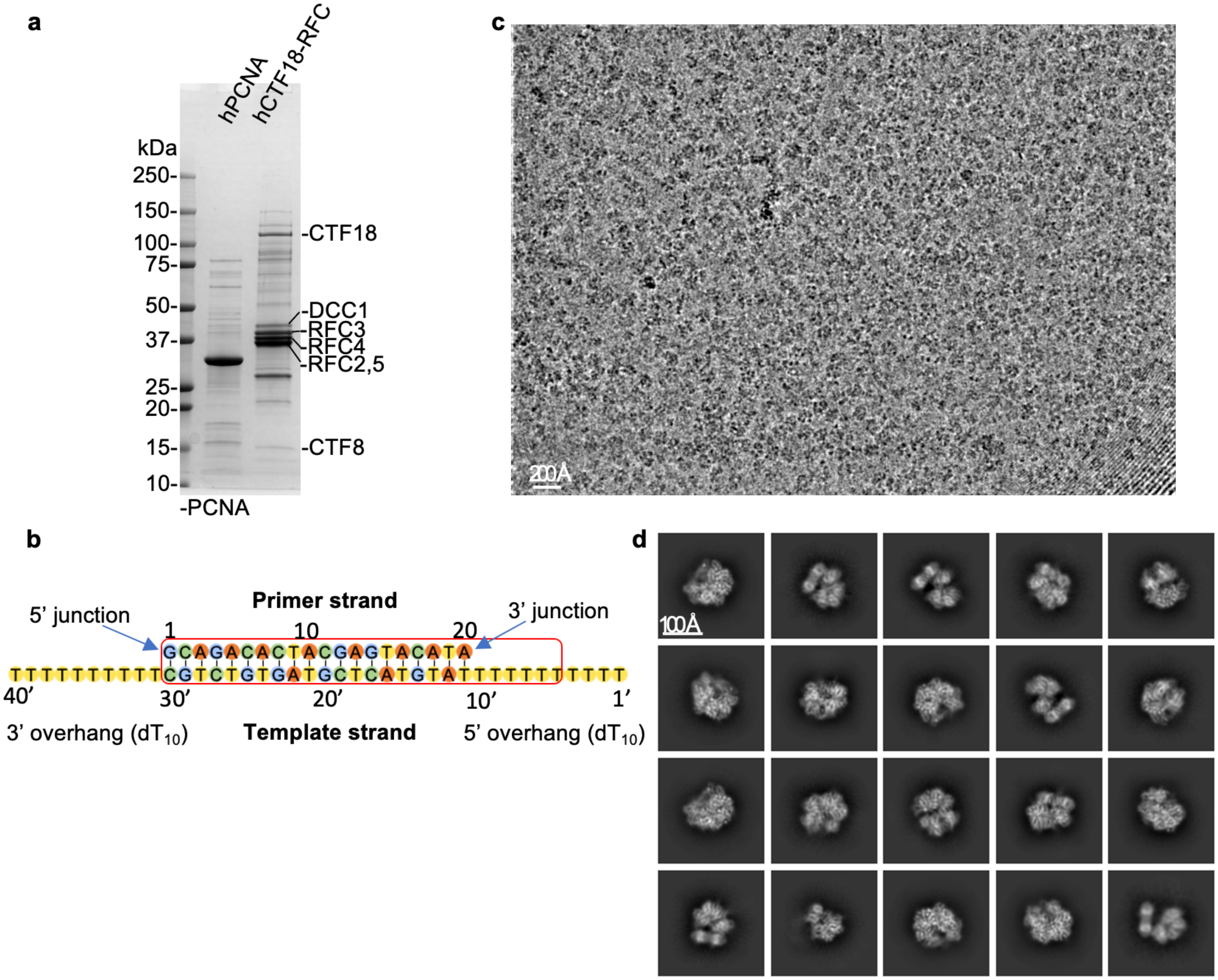

SI Appendix, Fig. S1. Cryo-EM of in vitro assembled human CTF18-RFC–PCNA–DNA complex. a) Coomassie Blue stained SDS-PAGE gel (4-20%) of human PCNA and CTF18-RFC. **b**) The double-tailed DNA substrate used in this study. The 5′- and 3′-recessed DNA junctions are labeled. Nucleotides in the red box are resolved in the EM maps. **c**) A representative raw micrograph of the mixture of CTF18-RFC, PCNA, and DNA substrate. A total of 18,239 micrographs were recorded. **d**) Selected 2D class averages of the particle images of the human CTF18-RFC–(DNA)–PCNA complexes.

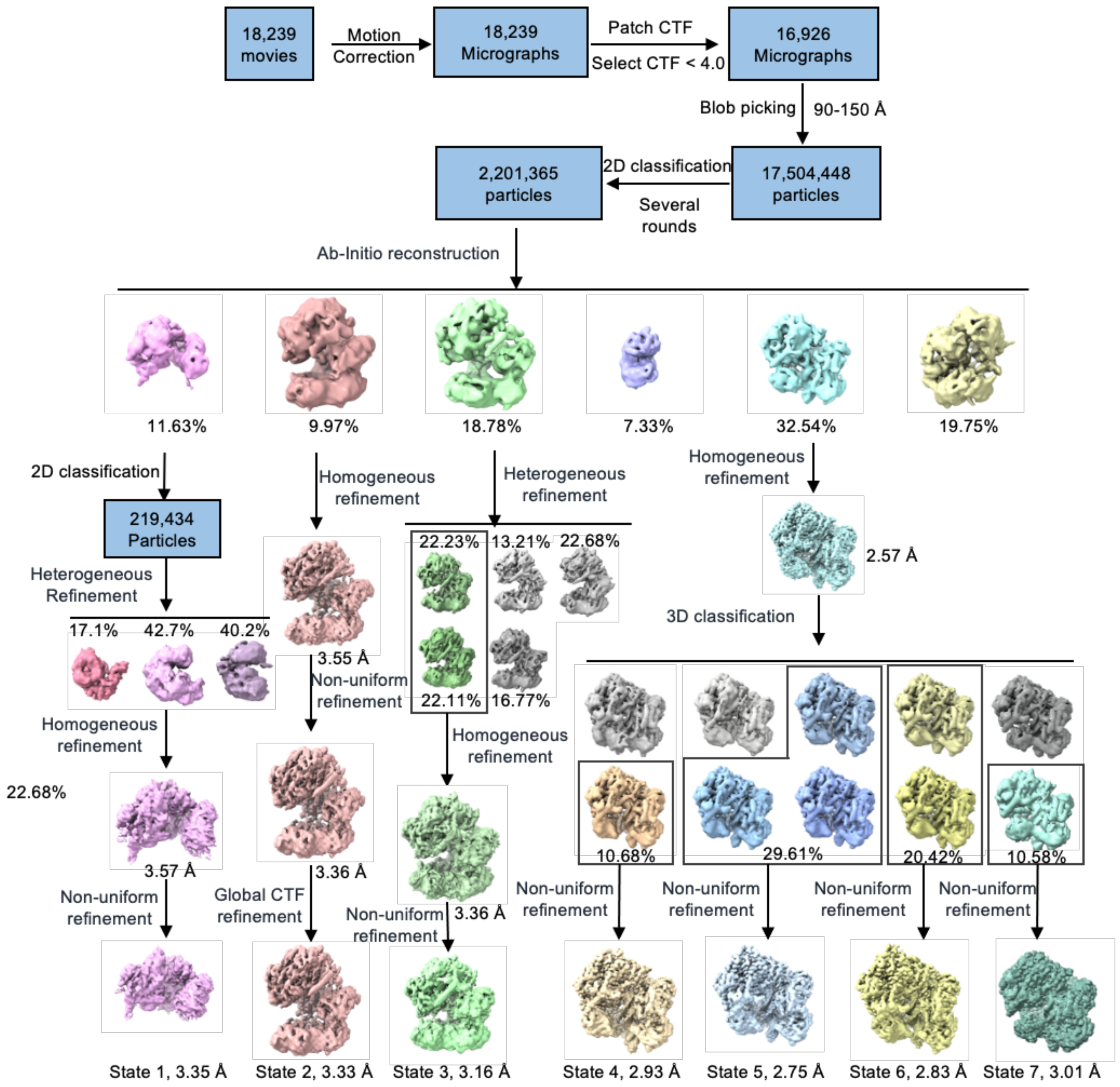

SI Appendix Fig. S2. Workflow of cryo-EM image processing and 3D reconstruction. CryoSPARC (version 4.2.1) was used for image processing and 3D reconstruction. 2D classification, 3D classifications, and 3D reconstructions and refinements resulted in seven 3D EM maps representing the CTF18-RFC complex alone, the binary CTF18-RFC–PCNA complexes, and the ternary CTF18-RFC–PCNA complexes.

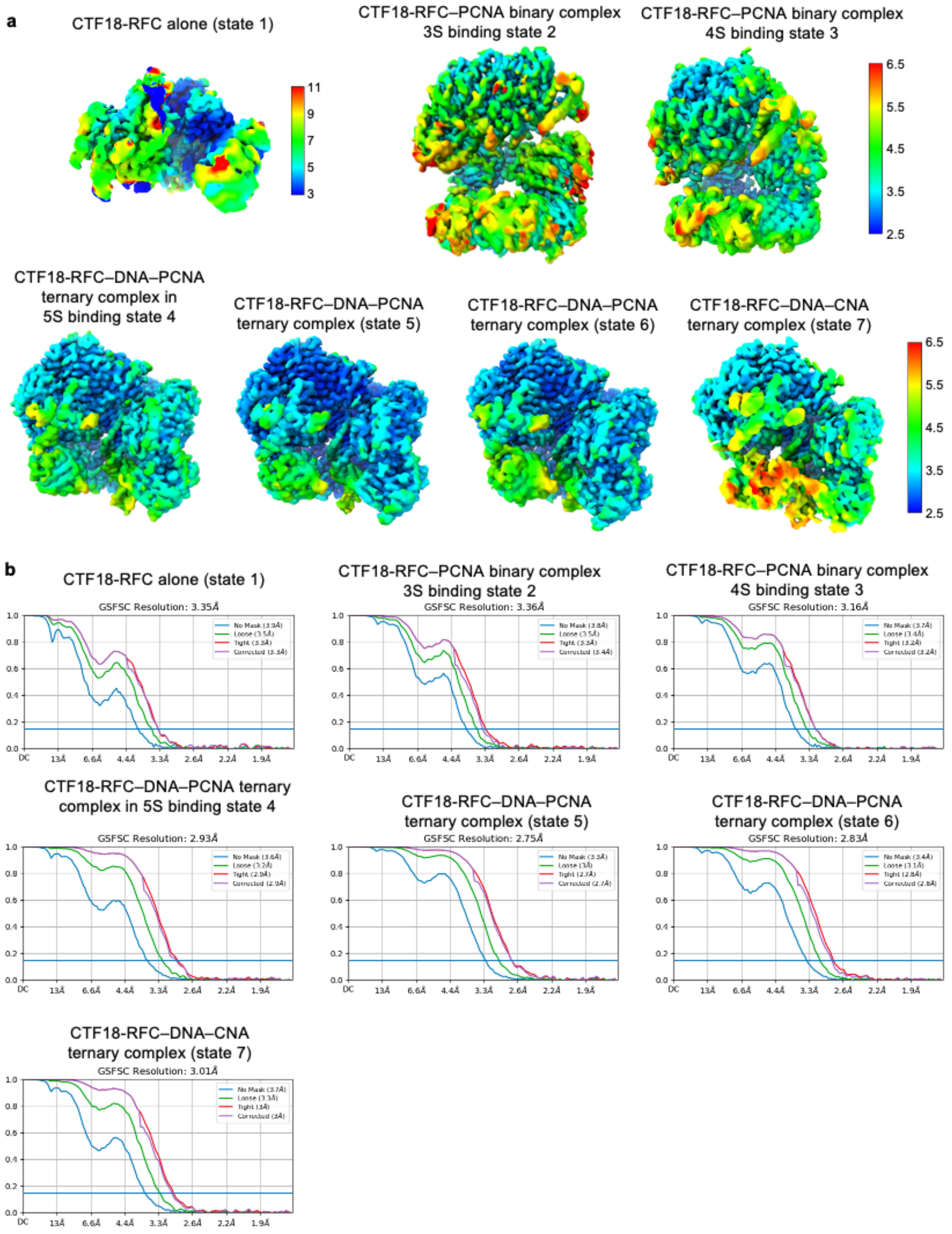

SI Appendix, Fig. S3. Resolution estimation of the seven EM maps of the human CTF18-RFC loading complexes. **a)** Color-coded local resolution maps of the seven EM maps of the CTF18-RFC loading complexes. **b)** Gold standard Fourier shell correlation (GSFSC) curves of the EM maps.

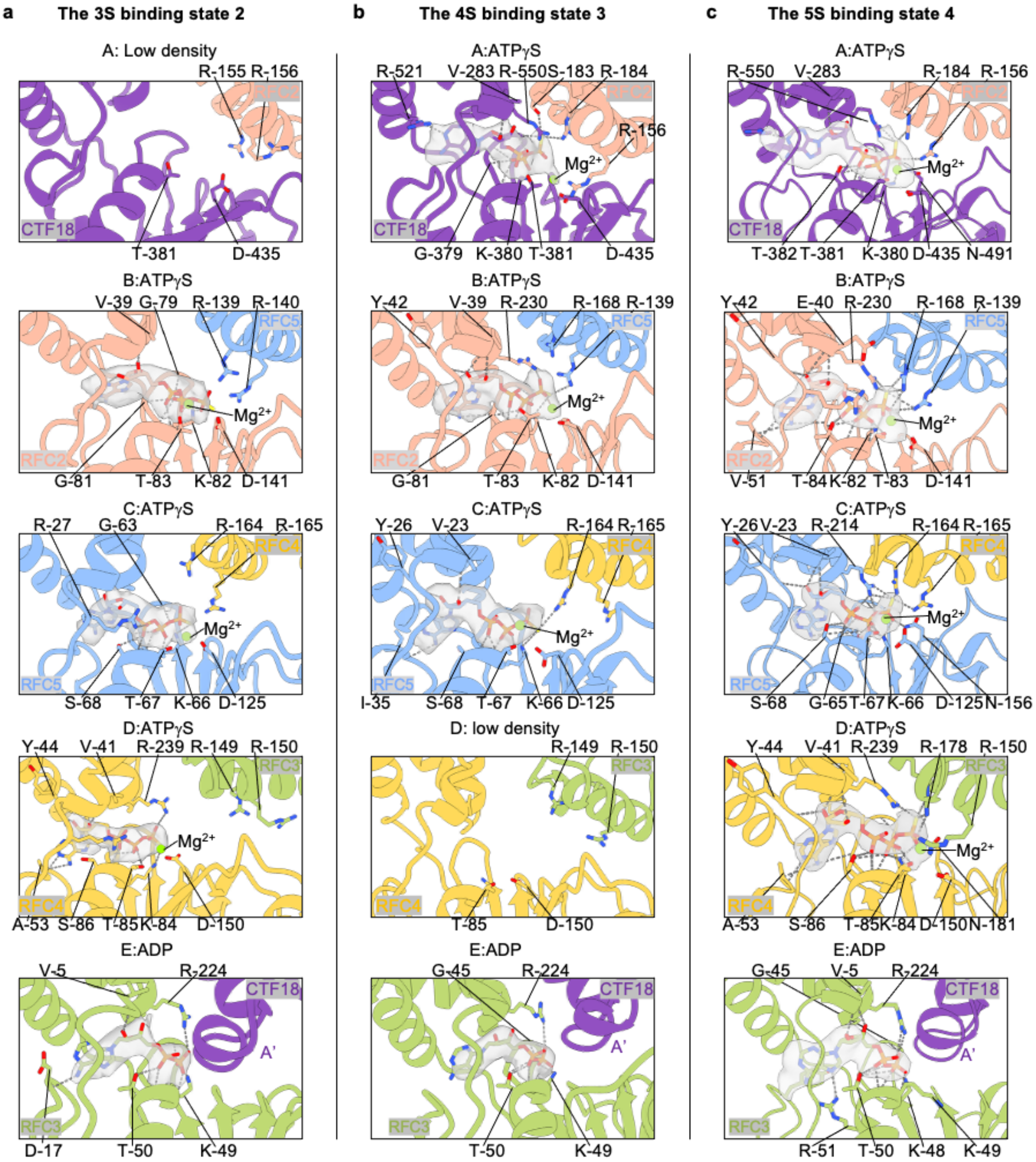

SI Appendix, Fig. S4. Nucleotide-binding in the 3S, 4S, and 5S binding states of the CTF18-RFC–PCNA complexes. a-c) The five nucleotide-binding sites in the 3S binding state 2 (a), the 4S binding state 3 (b), and the 5S binding state 4 (c). ATPγS and ADP are shown as sticks with their respective EM densities (in transparent gray surfaces) superimposed. The resolved Mg^{2+} ions are in green spheres. One EM map is surfaced and rendered at one threshold. Residues coordinating the nucleotides are shown in sticks and labeled.

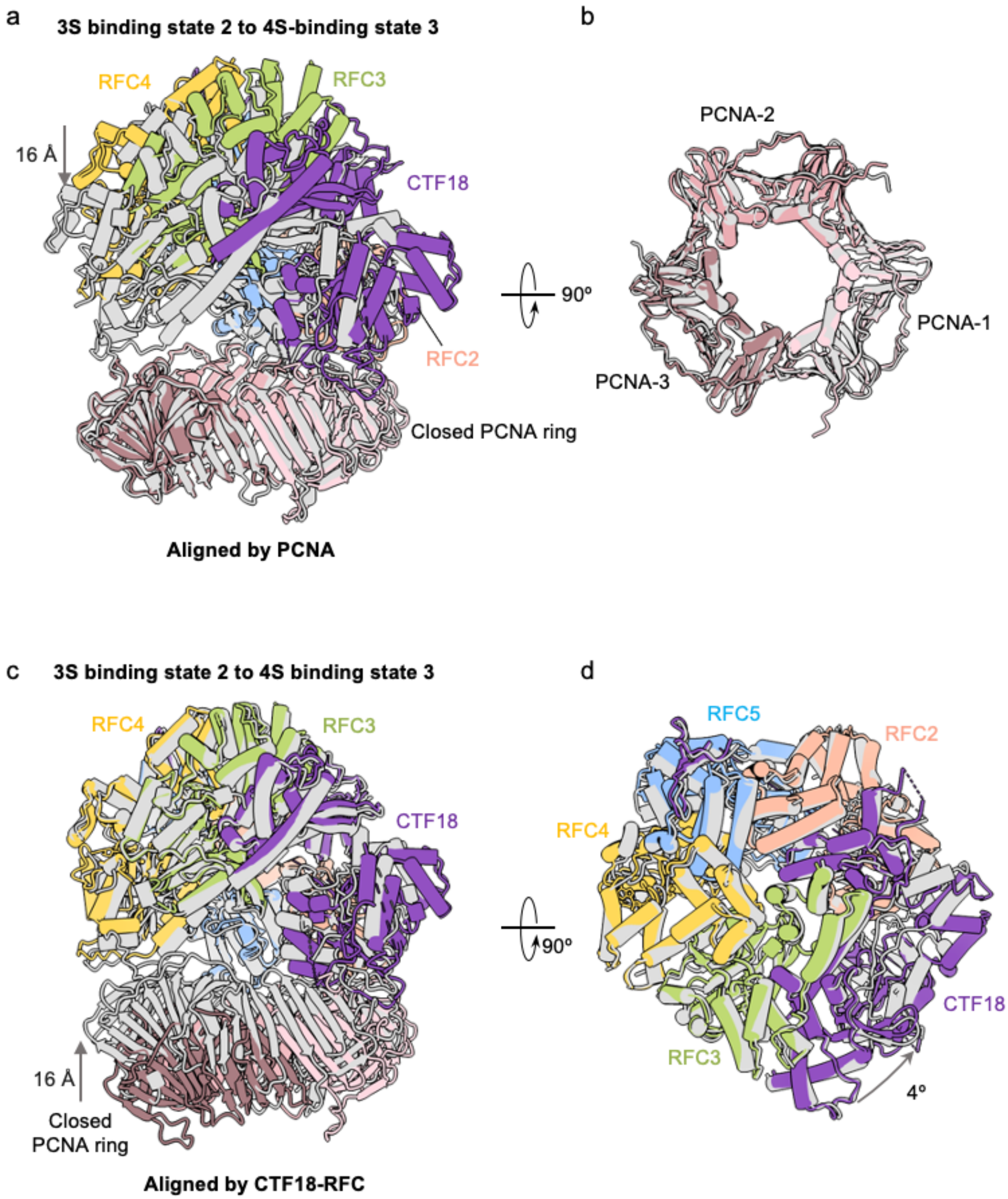

SI Appendix, Fig. S5. Comparison of the CTF18-RFC–PCNA structures in the 3S and 4S binding states. **a-b)** Side and top views of the structures of the 3S binding state 2 (color) and 4S binding state 3 (gray) aligned by the shared closed PCNA ring. CTF18-RFC was omitted in the right panel for clarity. **c-d**) Side and top views of the structures of the 3S binding (color) and 4S-binding (gray) states aligned by CTF8-RFC. Both CTF18-RFC and PCNA can be well superimposed, indicating that the conformational change from the 3S to the 4S binding state only involved rigid-body movements.

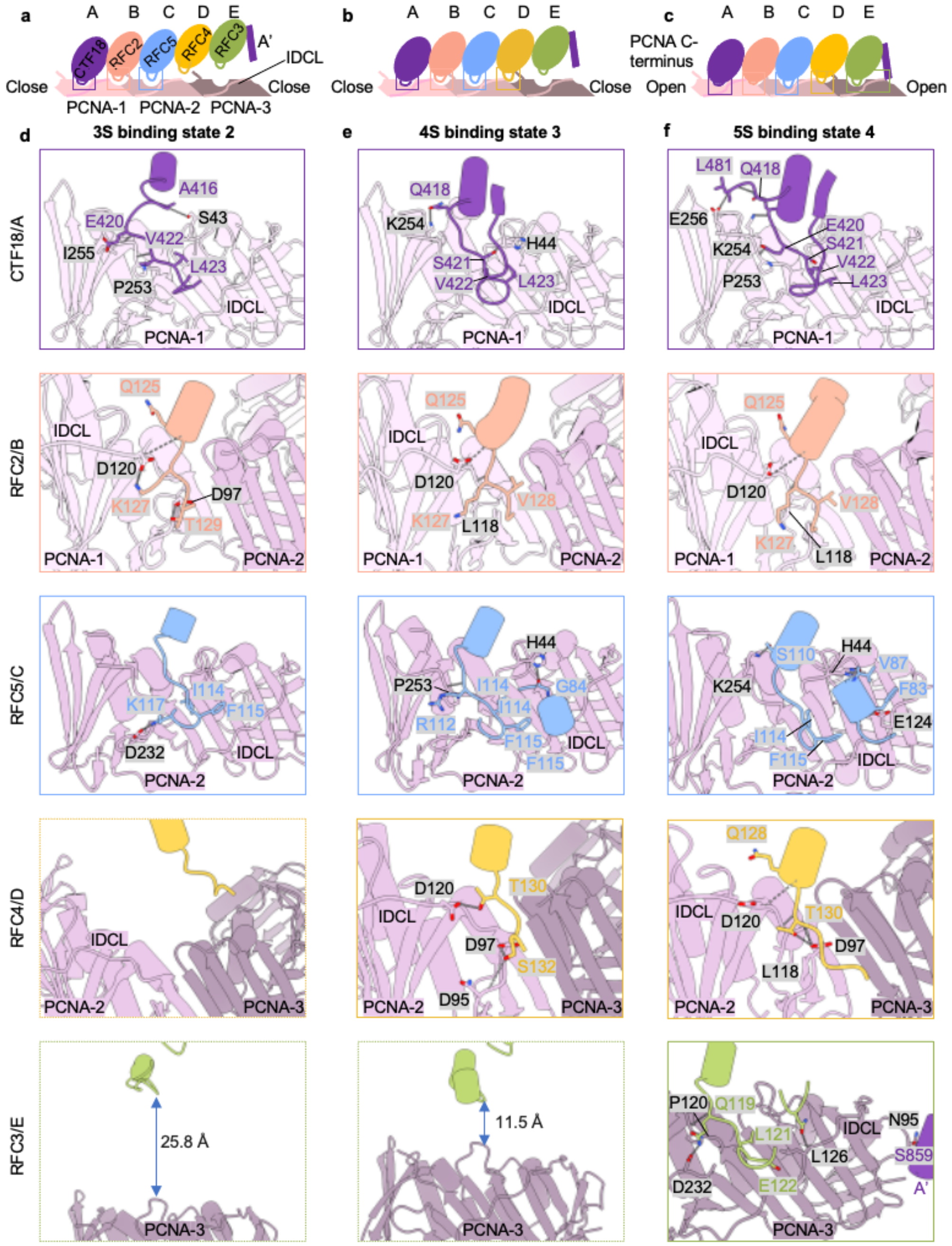

SI Appendix, Fig. S6. Contacts between PCNA and individual CTF18-RFC subunits in the 3S, 4S, and 5S binding states. **a-c)** Sketches of contacts between CTF18-RFC and PCNA in the 3S-binding state 2 (a), 4S binding state 3 (b), and 5S binding state 4 (c). PCNA and CTF18-RFC AAA+ ATPase modules are flattened for clarity. Key interacting residues are in sticks and labeled. Subunits A and C contact PCNA IDCL, and subunit B contacts the interface of PCNA-1 and PCNA-2 in a. Subunit D contacts the interface of PCNA-2 and PCNA-3 in b. Subunit E contacts the IDCL, and the CTF18 A'-domain contacts the N-terminus of PCNA-3 in c. **d-f**) Closeup views of contacts between CTF18-RFC and PCNA in the 3S (d), 4S (e), and 5S binding states (f). The CTF18 PIP motif inserts into the hydrophobic pocket of PCNA-1. The RFC5 PIP motif inserts into the hydrophobic PCNA-2 pocket. RFC3/E interacts with PCNA-3 hydrophobically via a non-PIP peptide. Subunits B and D do not bind the PCNA hydrophobic pocket but may form H-bonds with IDCL. The contact with PCNA gradually increases from the 3S to 4S to 5S binding states. RFC4/D establishes a new contact with PCNA-2 from 3S to 4S-binding state. RFC5/C and CTF18 A'-domain establish new contacts with PCNA-3 from the 4S to the S5 binding state.

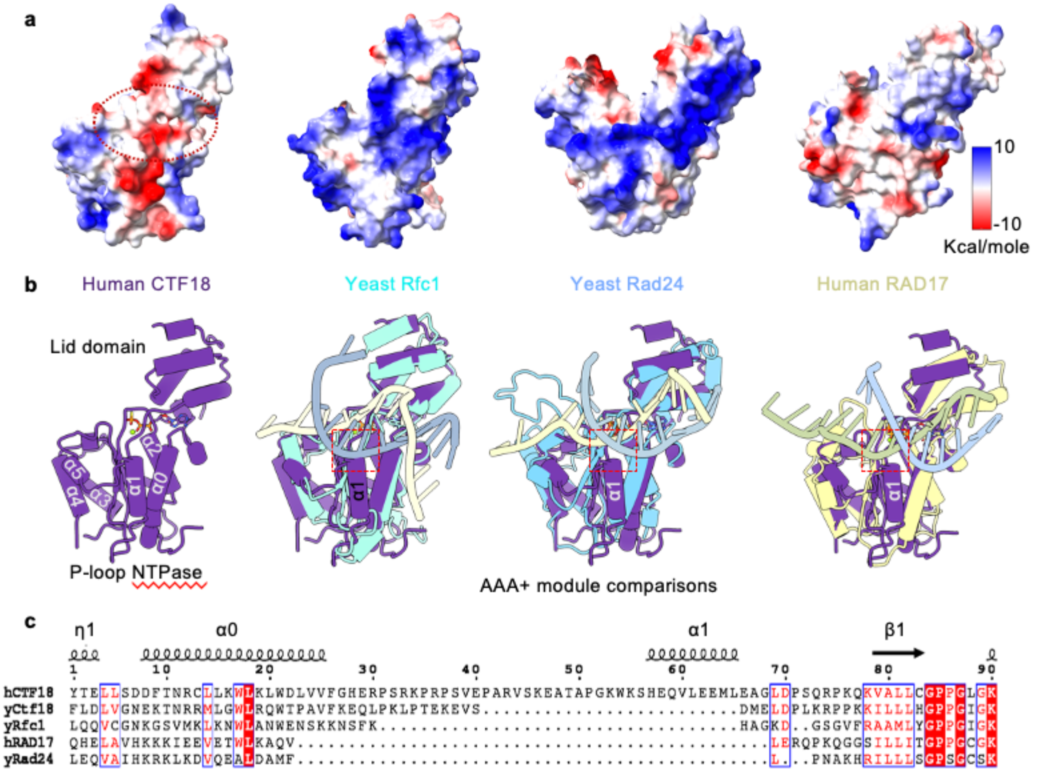

SI Appendix, Fig. S7. Structural comparison indicates that CTF18 doesn't have the external (shoulder) DNA binding site. a) Electrostatic surface potential of the A-gate-open human CTF18 AAA+ module in comparison with those of yeast Rfc1 and Rad24, and the human RAD17. Rfc1 and Rad24/RAD17 all contain a positively charged region for DNA binding at the external shoulder site, but the corresponding region in the human CTF18 is negatively charged as marked by a dashed red circle. **b**) The structures in cartoons correspond to the surface charge views in a, aligned by their respective α/β (Rossman) fold. Rfc1 and Rad24/RAD17 bind a dsDNA in their shoulder sites. The shoulder DNA in Rfc1 and Rad24/RAD17 would sterically clash with helix α1 of the CTF18 Rossman fold. **c**) Sequence alignment of the AAA+ modules of human CTF18, yeast Ctf18, yeast Rfc1, yeast Rad24, and human RAD17 at the α1-helix region, revealing the unique α1-helix in the human CTF18. The alignment was performed with ESPript 3.0 (https://espript.ibcp.fr).

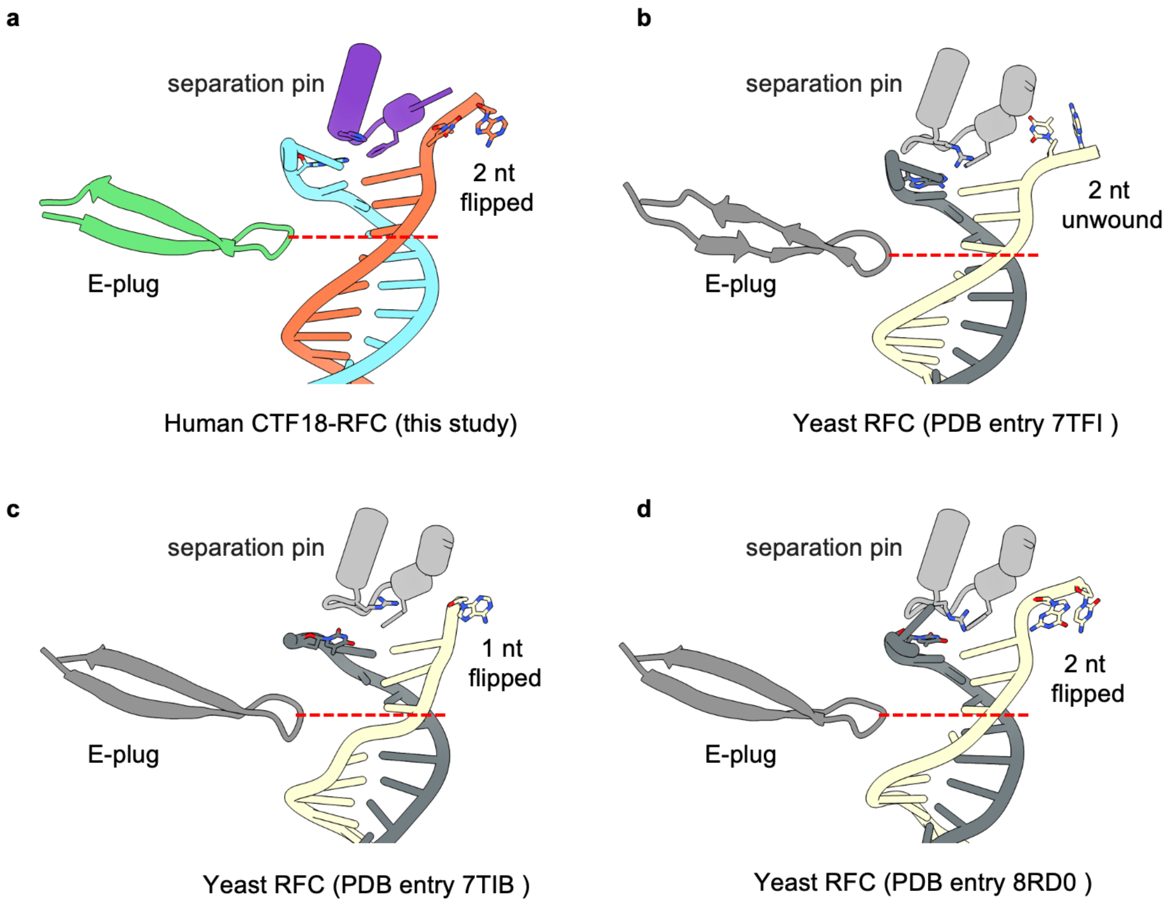

SI Appendix, Fig. S8. Comparison of separation pins and E-plug in the current human CTF18-RFC–DNA–PCNA structure and three published yeast RFC–DNA–PCNA structures. **a)** Close-up view of the interactions between CTF18-RFC and 3' ss/ds DNA junction in the ATPase chamber. **b-d)** Close-up view of the interactions between yeast RFC and three different DNA substrates all containing a 3'-ss/ds junction in the ATPase chamber. Because E-plug inserting into the DNA major groove (the dashed red lines) is a conserved feature of the clamp loaders, and the separation pin is 3 base pairs above the DNA major groove, any base pairs longer than 3 from the major groove (2, 1, 2, and 2 bp in a-d, respectively) are melted by the separation pin.

References for SI Appendix

1. F. Weissmann *et al.*, biGBac enables rapid gene assembly for the expression of large multisubunit protein complexes. *Proc. Natl. Acad. Sci. U.S.A.* **113**, E2564-E2569 (2016).
2. D. N. Mastronarde, Advanced data acquisition from electron microscopes with SerialEM. *Microsc. Microanal.* **24**, 864-865 (2018).
3. S. Q. Zheng *et al.*, MotionCor2: anisotropic correction of beam-induced motion for improved cryo-electron microscopy. *Nat. Methods* **14**, 331-332 (2017).
4. A. Punjani, J. L. Rubinstein, D. J. Fleet, M. A. Brubaker, cryoSPARC: algorithms for rapid unsupervised cryo-EM structure determination. *Nat. Methods* **14**, 290-296 (2017).
5. T. D. Goddard *et al.*, UCSF ChimeraX: Meeting modern challenges in visualization and analysis. *Protein Sci.* **27**, 14-25 (2018).
6. P. Emsley, K. Cowtan, Coot: model-building tools for molecular graphics. *Acta Crystallogr. D* **60**, 2126-2132 (2004).
7. P. D. Adams *et al.*, PHENIX: a comprehensive Python-based system for macromolecular structure solution. *Acta Crystallogr. D* **66**, 213-221 (2010).
8. C. J. Williams *et al.*, MolProbity: More and better reference data for improved all-atom structure validation. *Protein Sci.* **27**, 293-315 (2018).
9. R. Sanchez-Garcia *et al.*, DeepEMhancer: a deep learning solution for cryo-EM volume post-processing. *Commun. Biol.* **4**, 874 (2021).
